# Gene network centrality analysis identifies key regulators coordinating day-night metabolic transitions in *Synechococcus elongatus* PCC 7942 despite limited accuracy in predicting direct regulator-gene interactions

**DOI:** 10.3389/fmicb.2025.1569559

**Published:** 2025-03-26

**Authors:** Zachary Johnson, David Anderson, Margaret S. Cheung, Pavlo Bohutskyi

**Affiliations:** ^1^Biological Sciences Division, Earth and Biological Sciences Directorate, Pacific Northwest National Laboratory, Richland, WA, United States; ^2^Department of Biological Systems Engineering, Washington State University, Pullman, WA, United States; ^3^Environmental Molecular Sciences Laboratory, Pacific Northwest National Laboratory, Richland, WA, United States; ^4^Department of Physics, University of Washington, Seattle, WA, United States

**Keywords:** gene regulatory networks, circadian regulation, gene network centrality analysis, regulation of day-night transition, coordination of temporal metabolism, network-based key regulator discovery, regulators for circadian-optimized bioproduction

## Abstract

*Synechococcus elongatus* PCC 7942 is a model organism for studying circadian regulation and bioproduction, where precise temporal control of metabolism significantly impacts photosynthetic efficiency and CO_2_-to-bioproduct conversion. Despite extensive research on core clock components, our understanding of the broader regulatory network orchestrating genome-wide metabolic transitions remains incomplete. We address this gap by applying machine learning tools and network analysis to investigate the transcriptional architecture governing circadian-controlled gene expression. While our approach showed moderate accuracy in predicting individual transcription factor-gene interactions - a common challenge with real expression data - network-level topological analysis successfully revealed the organizational principles of circadian regulation. Our analysis identified distinct regulatory modules coordinating day-night metabolic transitions, with photosynthesis and carbon/nitrogen metabolism controlled by day-phase regulators, while nighttime modules orchestrate glycogen mobilization and redox metabolism. Through network centrality analysis, we identified potentially significant but previously understudied transcriptional regulators: HimA as a putative DNA architecture regulator, and TetR and SrrB as potential coordinators of nighttime metabolism, working alongside established global regulators RpaA and RpaB. This work demonstrates how network-level analysis can extract biologically meaningful insights despite limitations in predicting direct regulatory interactions. The regulatory principles uncovered here advance our understanding of how cyanobacteria coordinate complex metabolic transitions and may inform metabolic engineering strategies for enhanced photosynthetic bioproduction from CO_2_.

## 1 Introduction

The field of synthetic and systems biology faces a critical challenge: extracting meaningful biological knowledge about gene expression regulation from the overwhelming volume of RNA-sequencing and other omics data. This challenge is particularly acute in photosynthetic organisms, which must orchestrate complex metabolic transitions between day and night cycles through multilayered regulation. Understanding this temporal control is crucial for both fundamental biology and biotechnology applications. *Synechococcus elongatus* PCC 7942 (PCC 7942) serves as a key model organism for studying circadian regulation and a platform for sustainable bioproduction from CO_2_. During daytime, circadian-driven regulation upregulates photosynthesis and Calvin-Benson cycle activities, channeling excess reducing power into glycogen storage, nitrate reduction, and bioproduct synthesis (Liu et al., 2012; Mullineaux, 2014; Hudson, 2023; Grund et al., 2019; Abramson et al., 2016; Santos-Merino et al., 2024). At night, circadian control rewires metabolism, upregulating glycogen breakdown and reducing equivalent generation through the oxidative pentose phosphate pathway (OxPPP) (Welkie et al., 2019; Shinde et al., 2020). The significance of these circadian regulatory mechanisms - and the importance of understanding them - is highlighted by their ability to modulate photosynthetic productivity up to threefold through coordinated control of carbon metabolism and competing cellular processes (Gilliam et al., 2025).

Gene Regulatory Networks (GRNs), particularly when integrated with genome-scale metabolic models (GSMs), have emerged as powerful tools for analyzing complex biological data and enabling refined control of cellular phenotypes (Lee and Kim, 2015; Ko et al., 2020; Choi et al., 2019; Yilmaz et al., 2022). While established regulatory databases like RegulonDB (Huerta et al., 1998; Salgado et al., 2024; Tierrafría et al., 2022) and YEASTRACT+ (Abdulrehman et al., 2011; Teixeira et al., 2018; Teixeira et al., 2023) showcase successful network mapping in *E. coli* and *S. cerevisiae*, developing similar understanding for photosynthetic organisms remains challenging due to their complex light-dependent metabolism and circadian control systems.

Briefly, the core circadian KaiABC clock regulates metabolic transitions through 24-h oscillations in KaiC phosphorylation (Ishiura et al., 1998; Xu et al., 2000). KaiC controls two histidine kinases - SasA (kinase) and CikA (phosphatase) – which regulate phosphorylation state of the master regulator RpaA (Gutu and O’Shea, 2013; Taniguchi et al., 2010) driving genome-wide oscillations in gene expression (Markson et al., 2013; Puszynska and O'Shea, 2017). A second global regulator RpaB controls photosynthesis and oxidative stress (Vijayan et al., 2009; Nishiwaki et al., 2004; Markson et al., 2013) while also targeting promoters of RpaA, KaiB, and KaiC, thus linking to the core circadian clock (Hanaoka et al., 2012; Piechura et al., 2017). Additionally, several circadian-dependent sigma factors act as global co-regulators by directing RNA polymerase (Fleming, 2017; Fleming and O’Shea, 2018). Genome-wide DNA binding sites have been identified by ChIP-seq for key circadian regulators in PCC 7942, including RpaA and RpaB, and the sigma factors RpoD5, RpoD6, and SigF2 (Piechura et al., 2017; Markson et al., 2013; Fleming, 2017; Fleming and O’Shea, 2018). While the core clock components and several global regulators are well-characterized, a critical knowledge gap remains in how secondary regulatory elements link circadian oscillators to genome-wide metabolic transitions. Deciphering these regulatory components and their contribution to diurnal metabolic control is essential for both basic research and metabolic engineering of photosynthetic cell factories. However, mapping these regulatory networks in cyanobacteria presents unique challenges due to their complex light-dependent metabolism and multilayered circadian control systems.

Traditional approaches to mapping GRNs focus on predicting direct transcription factor-gene (TF-gene) interactions, but accurate prediction remains challenging despite algorithmic advances. The DREAM5 network inference challenge demonstrated that even top-performing methods like GENIE3 (Huynh-Thu et al., 2010) achieve only modest accuracy on synthetic benchmark data with highest precision-recall (AUPR) of ~0.3 (Marbach et al., 2012). Performance drops significantly with real gene expression data, particularly in complex organisms - prediction accuracy for TF-gene interactions in *E. coli* typically shows AUPR values of only 0.02–0.12 (Marbach et al., 2012; Escorcia-Rodríguez et al., 2023). Integration of additional data types (protein-DNA interactions, gene functions, DNA topology-dependent accessibility) and advanced computational methods has yielded only incremental improvements (Iglesias-Martinez et al., 2021; Häusler, 2024; Passemiers et al., 2022; Razaghi-Moghadam and Nikoloski, 2020; Zhao et al., 2021; Escorcia-Rodríguez et al., 2023).

These consistently modest accuracies, even in well-studied organisms with extensive validation data, likely reflect inherent complexity of transcriptional regulation. However, while GRNs show limited accuracy in predicting individual TF-gene interactions, they successfully capture higher-order regulatory patterns - network topology analysis reveals biologically meaningful gene modules, regulatory hierarchies, and functional communities that align with experimental observations (Sorrells and Johnson, 2015; Jothi et al., 2009; Bhardwaj et al., 2010; Fang et al., 2017). This network-level understanding is particularly valuable for photosynthetic organisms where temporal coordination of metabolism directly impacts cellular productivity. Based on this utility of network analysis, we applied GENIE3 to investigate how circadian regulatory architecture orchestrates metabolic transitions and carbon allocation - knowledge essential for understanding and engineering efficient photosynthetic cell factories.

Through integration of machine learning with network topology analysis, we demonstrate how biological insights can be extracted from high-throughput omics data. While individual regulatory predictions show limited accuracy, the network’s emergent properties – topology, community structure, and centrality patterns – reveal biologically meaningful organization. Through analysis of network centrality metrics in the context of circadian expression patterns, we identify distinct regulatory modules coordinating day/night metabolism and highlight previously uncharacterized regulators of metabolic transitions. Our findings demonstrate how network-level analysis can extract valuable insights despite uncertainty in direct TF-gene predictions, advancing both fundamental understanding of cyanobacterial regulation and providing a framework applicable to other organisms. Uncovering these regulatory mechanisms has direct implications for engineering metabolically efficient photosynthetic cell factories while contributing to our knowledge of circadian control in transition between day and night metabolism.

## 2 Methods

This section details our systematic approach to inferring and analyzing transcriptional regulatory networks governing circadian-regulated metabolic processes in *S. elongatus* PCC 7942.

### 2.1 Construction and quality control of multi-source gene expression dataset

Raw RNA-Seq data was acquired as of January 31, 2023, from three major repositories: the NCBI Sequence Read Archive (SRA) (Katz et al., 2022), Gene Expression Omnibus (GEO) (Barrett et al., 2013), and Joint Genome Institute (JGI) (Nordberg et al., 2014). The reads were mapped against the following reference sequences: chromosome (NC_007604.1), pANL plasmid (NC_004073.2), and pANS plasmid (NC_004990.1).

Quality control was performed in multiple stages. Initial assessment using FastQC (Andrew, 2010) was followed by manual curation to select samples with sufficient experimental metadata. Low-quality samples were filtered using stringent criteria, including removal of samples with fewer than 100,000 total reads. The data was then log-transformed to TPM values, followed by evaluation of global correlation between replicates. Samples with correlation coefficients below 0.9 between replicates were removed. For time-series datasets without biological replicates, we applied sliding window correlation between adjacent timepoints.

The final curated dataset (named selongEXPRESS) consisted of 330 samples with log-TPM transformed gene counts. Complete sample metadata, quality control metrics, normalized expression values, and gene annotation are provided in Supplementary Tables S1–S4.

### 2.2 Multi-method approach for gene regulatory network inference

We employed three complementary computational approaches to predict transcription factors (TFs) in PCC 7942: (i) Predicted Prokaryotic Transcription Factors (P2TF) database (Ortet et al., 2012), (ii) Encyclopedia of Well-Annotated DNA-binding Transcription Factors (ENTRAF) (Ledesma et al., 2022), and (iii) deep learning-based DeepTFactor (Kim et al., 2021). These pipelines combine knowledge from established transcriptional regulation databases [RegulonDB on *E. coli* (Salgado et al., 2024), on *B. subtilis* (Sierro et al., 2008), UniProt (The UniProt Consortium, 2023), DNA-binding domain database (Wilson et al., 2008)] with sequence-based prediction methods using hidden Markov models (Mistry et al., 2021) and convolutional neural networks (Kim et al., 2021). The complete list of predicted TF candidates is provided in Supplementary Table S5.

For quantifying TF-to-gene expression associations, we used the random forest-based ensemble algorithm GENIE3 (Huynh-Thu et al., 2010). The algorithm was constrained by coupling the PCC 7942 gene expression matrix with the unified set of predicted DNA-binding TF candidates from all three prediction pipelines. The resulting GRN was represented as a matrix of 331,977 values corresponding to predicted regulatory interaction strengths between 123 predicted TF candidates and 2,700 genes.

**Figure 1:**
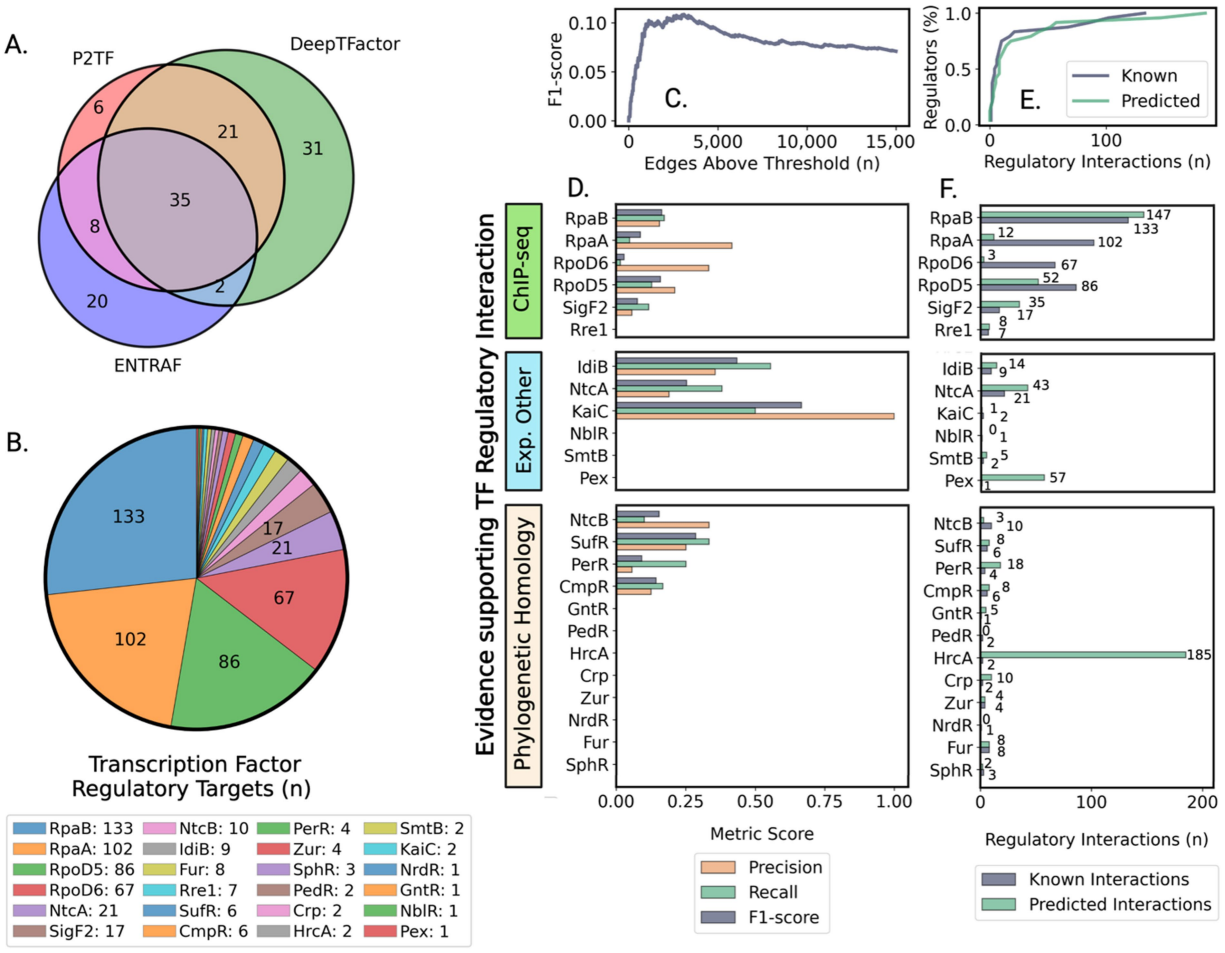
Assessment of transcription factor prediction and regulatory network accuracy reveals biologically consistent patterns despite moderate precision. **(A)** Venn diagram showing overlapping sets of transcription regulators identified by three computational prediction methods: [P2TF (Ortet et al., 2012), ENTRAF (Ledesma et al., 2022), and DeepTFactor (Kim et al., 2021)]. **(B)** Number of known regulatory interactions for a subset of characterized regulators based on literature and experimental studies that provided a validation framework for network predictions. **(C)** Optimization of network size to balance prediction accuracy with comprehensive regulatory coverage using F1-score metrics. **(D)** Evaluation of prediction accuracy for different types of transcriptional regulators grouped by evidence source, showing higher accuracy for well-characterized regulatory systems. **(E)** Network successfully captures the biological distribution of regulatory interactions, where most genes are controlled by few regulators. **(F)** Strong correlation between predicted and experimentally validated regulatory targets, grouped by evidence source, demonstrates biological relevance of network predictions.

To focus our analysis on TF-gene interactions with the highest biological significance, we reduced the network to include only edges with the strongest predicted regulatory weights. The GRN size was optimized to 3,102 edges by selecting an edge cutoff that maximized the micro-average F1-score when evaluated against experimentally validated TF-gene interactions (Supplementary Table S6). The validation set comprised regulatory interactions for 24 previously characterized TFs with strong experimental evidence (detailed in section 3.1 and Figure 1). The F1-score was computed using Equations 1.1–1.3, with each TF evaluated as a binary classifier at each edge cutoff threshold. Specifically, *TP_ni_* corresponds to the overlap between predictions and known interactions at edge cutoff i for TF_n_. *FN_ni_* represents the number of known interactions missing from our predictions for TF_n_ at the edge cutoff. FP_n_ indicates the number of interactions predicted by our approach but not present in the set of experimentally validated regulatory interactions at the edge cutoff. After optimization, we removed 12 edges not connected to the primary connected component, resulting in a final network comprising 1,839 nodes and 3,090 edges (Supplementary Table S7).


1.1 $$\mathrm{Pr}e_{\mathrm{micro}\;\left(i\right)}=\frac{\left(TP_{1i}+TP_{2i}+\ldots +TP_{ni}\right)}{\left(TP_{1i}+TP_{2i}+\ldots +TP_{ni}\right)+\left(FP_{1i}+FP_{2i}+\ldots +FP_{ni}\right)}$$

1.2 $$Rec_{\mathrm{micro}\;\left(i\right)}=\frac{\left(TP_{1i}+TP_{2i}+\ldots +TP_{ni}\right)}{\left(TP_{1i}+TP_{2i}+\ldots +TP_{ni}\right)+\left(FN_{1i}+FN_{2i}+\ldots +FN_{ni}\right)}$$

1.3 $$F1_{\mathrm{micro}\;\left(i\right)}=2\times \frac{\mathrm{Pr}e_{\mathrm{micro}\;\left(i\right)}*Rec_{\mathrm{micro}\;\left(i\right)}}{\mathrm{Pr}e_{\mathrm{micro}\;\left(i\right)}+Rec_{\mathrm{micro}\;\left(i\right)}}$$

### 2.3 Network topology and centrality analysis for identifying hierarchical organization of transcriptional regulators

GRN properties and node centrality metrics were calculated using Networkx (Hagberg et al., 2008). Network connectivity was characterized through weakly connected components (WCC): subsets of nodes connected regardless of edge direction; strongly connected components (SCC): subsets connected when accounting for edge direction; network density (Equation 2); average TF out-degree (number of genes targeted by each TF); and average gene in-degree (number of TFs regulating each gene).

The following centrality metrics were used to assess transcription factor importance:

#### 2.3.1 Network density: quantifying global connectivity


2 $$d=\frac{m}{n\left(n-1\right)}$$

where n is the number of nodes and m is the number of edges.

#### 2.3.2 Degree centrality: measuring direct regulatory interactions


3 $$d\left(v\right)=|V|$$

where |V| is the number of neighbors directly connected to node v. For our GRN, DC represents the number of predicted TF-gene regulatory interactions for a specific TF (Newman, 2008).

#### 2.3.3 Betweenness centrality: identifying information flow mediators

BC is based upon the concept of shortest pathways in a network and defines the fraction of shortest paths than a given node falls on between every pair of nodes, representing a measure of information flow through a given node (Freeman, 1977).


4 $$C_{B}\left(v\right)=\underset{s,t\in V}{\sum }\frac{\sigma \left(s,t|v|\right)}{\sigma \left(s,t\right)}$$

where *σ*(s, t) is the total number of shortest paths between nodes s and t, and σ(s, t|v) is the number of those paths passing through node v. Based on the concept of shortest network pathways, this metric quantifies how frequently a node mediates information flow between other nodes in the network (Freeman, 1977).

#### 2.3.4 Closeness centrality: assessing global influence range


5 $$C\left(u\right)=\frac{n-1}{\sum_{v=1}^{n-1}d\left(v,u\right)}$$

where d(v, u) is the shortest-path distance between nodes u and v, and n-1 is the number of reachable nodes from node u. This metric quantifies how quickly a node can reach all other nodes in the network through shortest paths (Freeman, 1978).

#### 2.3.5 Eigenvector centrality: evaluating connection quality


6 Ax=λx

where A is the adjacency matrix, *λ* is the largest eigenvalue, and x is the corresponding eigenvector. This metric quantifies node importance based on its connections to other influential nodes (Newman, 2008).

#### 2.3.6 K-core: detecting densely connected regulatory modules

Identifies the largest subgraph where each node has at least k connections to other nodes within that subgraph. K-core values are calculated by iteratively removing vertices with fewer than k connections until all remaining vertices have at least k connections within the subgraph (Batagelj and Zaversnik, 2003). While not a direct centrality measure, k-core analysis has proven valuable for identifying protein complexes and functional modules in biological networks (Altaf-Ul-Amine et al., 2003; Kong et al., 2019).

#### 2.3.7 Mann–Whitney U test for differentiating centrality values of global versus local transcription factors

To determine whether high-impact transcription factors showed significantly higher centrality measures compared to local transcription factors, we employed the non-parametric Mann–Whitney U test. This test was selected for its suitability with small sample sizes and robustness against non-normal distributions. The null hypothesis tested whether the centrality measures had the same distribution for both global and local TFs, with the alternative hypothesis that global TFs showed higher centrality values. Statistical significance was assessed at *α* = 0.05.

### 2.4 Analysis of regulatory modules coordinating day-night metabolic transitions

To understand regulatory mechanisms governing day-night metabolic transitions crucial for both fundamental science and biotechnology applications, we mapped circadian expression peaks from an RNA-seq dataset (Vijayan et al., 2009) onto our inferred gene regulatory network. This integration of temporal expression data with network topology allowed us to investigate how transcriptional regulation orchestrates diurnal metabolic shifts.

#### 2.4.1 Hierarchical analysis of transcription factor target overlap

Transcription factor target overlap was characterized using unweighted pair group method with arithmetic mean (UPGMA) hierarchical clustering based on Jaccard distance. TF target gene sets were defined as the successors of each TF node using NetworkX (Hagberg et al., 2008). The Jaccard distance was computed for all pairwise sets of TF gene targets, with average-linkage used to define the hierarchical clusters of TF-TF-gene target overlap. Network modules under similar regulatory control were identified using Louvain community detection implemented in NetworkX, optimizing modularity within each community.

#### 2.4.2 Identification of co-regulated gene communities governing metabolic transitions

Communities of genes under coordinated regulation were identified using the Louvain community detection algorithm in NetworkX. This approach optimized modularity to reveal groups of genes likely to be controlled by similar regulatory mechanisms.

#### 2.4.3 Mapping regulatory communities to diurnal expression patterns

Only communities with more than 50% of genes attributed to a specific circadian phase (day or night) were considered for timing analysis, excluding non-circadian genes. The circular mean peak circadian time for each qualifying cluster was calculated using the *circmean* function from the *scipy.stats* module (Virtanen et al., 2020).

#### 2.4.4 Functional analysis of regulatory modules orchestrating transitions between day and night metabolism

Enrichment analysis employed Fisher’s exact test (Equation 7) with false-discovery rate (FDR) correction using the Benjamini-Hochberg procedure for Q-values:


7 $$p=\frac{\left(a+b\right)!\left(c+d\right)!\left(a+c\right)!\left(b+d\right)!}{a!b!c!d!n!}$$

where ***a*** – a number of genes in a module overlapping with the functional gene set; ***b*** – a number of genes in the module not in the predicted gene sets; ***c*** – a number of genes in the background gene set not in the predicted module; ***d*** – a background genes not in the predicted regulon or gene set.

Functional enrichments were determined using COG (Tatusov et al., 2001) and KEGG (Kanehisa and Goto, 2000) databases. COG enrichments were evaluated at q-value <0.05, while KEGG pathway enrichments used q-value <0.01.

## 3 Results

### 3.1 Network-level analysis reveals conserved architecture and overcomes limitations of direct TF-gene predictions

Before presenting our network analysis results, we highlight key assumptions underlying our approach. Our network construction combines multiple computational TF prediction methods to maximize coverage, with the GENIE3 random forest-based ensemble algorithm (Huynh-Thu et al., 2010) inferring regulatory relationships only based on gene expression dependencies and external specification of TFs. While we evaluate predictions against experimentally validated interactions, we recognize the inherent limitations in direct TF-gene prediction accuracy. To extract biologically meaningful patterns despite these limitations, we focus on network topology and centrality metrics, identifying significant regulatory elements based on their position in the network rather than relying solely on individual interaction predictions.

Gene regulatory network (GRN) reconstruction required integration of multiple computational approaches to address the complexity of transcriptional regulation in PCC 7942. We selected methods that complement each other’s strengths: sequence-based prediction of transcription factors, machine learning for pattern detection in large-scale expression data, and network topology analysis to reveal regulatory hierarchies.

**Figure 2:**
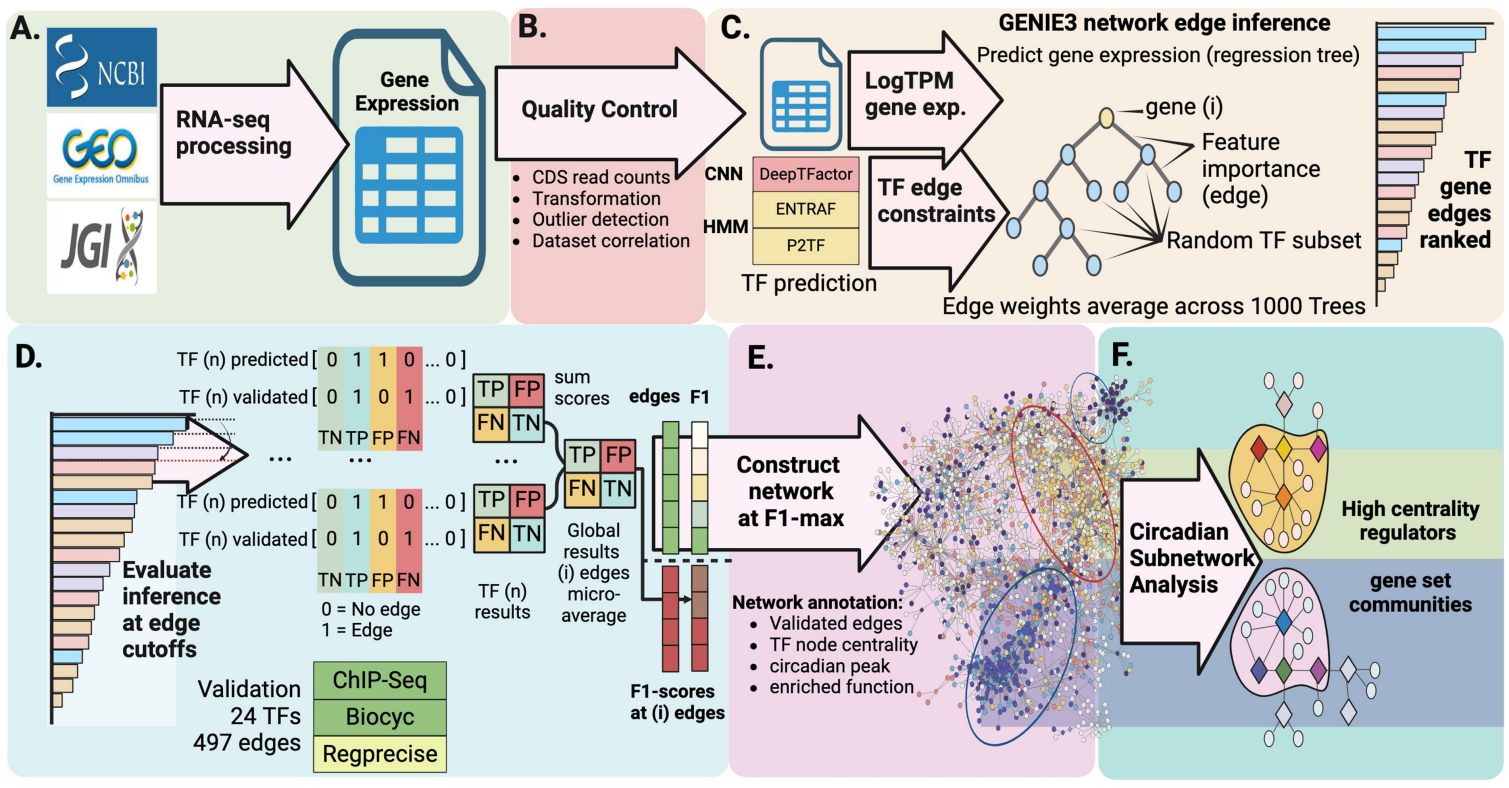
Computational pipeline for discovering circadian regulatory networks. **(A)** Collection and processing of publicly available RNA-seq datasets representing diverse physiological states and environmental conditions. **(B)** Data quality control and comprehensive identification of putative transcription factors through multiple prediction approaches. **(C)** Construction of gene regulatory network using GENIE3 machine learning algorithm to detect potential regulatory interactions. **(D)** Validation and refinement of predicted regulatory connections against known interactions. **(E)** Integration of network analysis with gene expression patterns to reveal regulatory modules controlling day-night metabolic transitions. **(F)** Characterization of circadian subnetworks revealing high-centrality regulators and functionally enriched gene set modules.

To implement this strategy, we developed an integrated computational pipeline that analyzes publicly available RNA-sequencing data to map the transcriptional landscape of PCC 7942 (Figures 2A-F). Quality control of RNA-seq data (Figure 2B, Methods section 2.1) yielded 330 samples representing 208 unique expression states under diverse conditions (Supplementary Tables S3, S4). Three complementary methods [P2TF (Ortet et al., 2012), ENTRAF (Ledesma et al., 2022), and DeepTFactor (Kim et al., 2021)] were used to identify potential transcription factors (TFs). DeepTFactor’s CNN classifier predicted the highest number of TFs (89), while the HMM-based methods P2TF and ENTRAF identified 70 and 65 TFs respectively, with notably greater agreement between DeepTFactor and P2TF predictions than with ENTRAF. To maximize coverage of potential regulatory interactions, we used an inclusive approach combining predictions from all three methods, resulting in 123 total potential TFs for network edge predictions (Figure 2C, Methods section 2.2, Supplementary Table S5), though only 35 TFs were predicted by all three methods (Figure 1A).

The GENIE3 machine learning algorithm, selected for its performance in DREAM 4 and 5 network inference challenges (Greenfield et al., 2010; Marbach et al., 2012), was applied to predict regulatory interactions between TFs and protein-coding genes. Network predictions were evaluated against a subset of 497 experimentally validated TF-gene interactions involving 24 TFs (Figures 1B, 2D; Supplementary Table S6). Most of these known regulatory connections came from ChIP-seq studies of global circadian regulators RpaA and RpaB, and sigma factors RpoD5, RpoD6, and SigF2 (Piechura et al., 2017; Markson et al., 2013; Fleming, 2017; Fleming and O’Shea, 2018), as well as from promoter validation (Kutsuna et al., 2007; Luque et al., 1994; Nakahira et al., 2004; Yousef et al., 2003; Morby et al., 1993; Kato et al., 2008; Karp et al., 2019; Xu et al., 2004; Ishiura et al., 1998), and phylogenetically conserved regulons predicted through promoter homology (Luque et al., 1994).

Given PCC 7942’s largely unmapped regulatory landscape, we approached validation as a multi-label classification task (Equations 1.1–1.3) (Pliakos and Vens, 2019; Ghamrawi and McCallum, 2005). The maximum micro-average F1-score of 0.11 was achieved at 3,102 edges (Figure 1C). Binary classification metrics for individual TF regulons grouped by evidence type are shown in Figure 1D. The network best captured regulons of IdiB (precision = 0.36, recall = 0.56) and NtcA (precision = 0.19, recall = 0.38). For global regulators involved into modulation of circadian cycle (RpaA, RpaB, RpoD5, RpoD6, and SigF2), average precision and recall were 0.23 ± 0.14 and 0.096 ± 0.06, respectively.

These accuracy metrics, while modest, reflect a common challenge in the field - for exceptionally well-studied organisms like *E. coli*, the prediction of TF-gene interactions from expression data typically achieves lower or comparable performance (Marbach et al., 2012; Escorcia-Rodríguez et al., 2023). Notably, the integration of additional data types such as protein-DNA interactions and DNA topology-dependent accessibility has yielded only modest improvements, with performance on real expression data remaining limited (Iglesias-Martinez et al., 2021; Häusler, 2024; Passemiers et al., 2022; Razaghi-Moghadam and Nikoloski, 2020; Zhao et al., 2021; Escorcia-Rodríguez et al., 2023). The challenge lies in capturing complex regulatory mechanisms that often involve indirect effects, cooperative binding, and temporal dynamics. However, despite limited accuracy in predicting individual interactions, the network’s global structure can reveal biologically meaningful patterns of regulation. Indeed, our network successfully modeled the distribution of known regulatory interactions (Figures 1E,F). The final connected component contained 3,090 edges analyzed as a directed graph (Supplementary Table S7). Network topology exhibited the sparsity characteristic of biological (Winterbach et al., 2013) and gene regulatory networks (Monteiro et al., 2020; Zorro-Aranda et al., 2022), with density of 9.1 × 10^−4^ (Equation 2). TFs averaged 27.8 ± 33.9 target genes (median = 14), while genes averaged 1.68 ± 0.89 regulating TFs (median = 1.00). This topology provided the foundation for using network centrality metrics to evaluate TF biological significance. Initial comparison between known high-impact regulators (RpaA, RpaB, RpoD5, RpoD6, SigF2, and master nitrogen regulator NtcA) and TFs governing local responses revealed significant differences in their network centrality metrics, prompting deeper investigation of centrality as an indicator of regulatory importance.

### 3.2 Network centrality metrics differentiate established global regulatory elements with system-wide influence from local transcriptional regulators

In biological networks, centrality metrics serve as quantitative indicators of a node’s functional importance and influence (Freeman, 1978; Freeman, 1977). For gene regulatory networks specifically, centrality measures have proven particularly valuable – highly central nodes often correspond to essential genes (del Rio et al., 2009; Park and Kim, 2009), members of key protein complexes (Kong et al., 2019), and regulators with global roles (Koschutzki and Schreiber, 2008; Naseri et al., 2021). We selected five complementary centrality metrics that capture different aspects of regulatory influence: degree centrality identifies regulators with many direct interactions, betweenness centrality highlights nodes that bridge different regulatory modules, closeness centrality reveals regulators that can rapidly influence the entire network, eigenvector centrality emphasizes connections to other influential regulators, and k-core detects regulators embedded in densely connected control modules (Equations 3-6).

We hypothesized that known global transcription factors would display higher centrality values compared to local response regulators. A one-sided Wilcoxon rank sum test confirmed this hypothesis across all metrics (*p* < 0.05; Table 1). Notably, eigenvector centrality and k-core proved most effective at distinguishing global from local regulators (*p* = 0.021 and 0.022 respectively), suggesting that influential transcription factors tend to form interconnected regulatory hubs rather than acting in isolation.

**Table 1:** Network analysis reveals distinct centrality patterns between global metabolic coordinators and pathway-specific local regulators in *S. elongatus* PCC 7942.

Category	TF	Degree	Betweenness (×10^−6^)	Closeness (×10^−3^)	Eigenvector (×10^−6^)	k-core
Cumulative Distribution	Mean	27.8	350	1.2	8,300	2.8
	SD	34.0	880	1.1	30,000	1.1
Cumulative Distribution	25%	5	0.0	0.27	1.0 × 10^−8^	2
	50%	14	12	1.1	1.9 × 10^−3^	3
	75%	37.5	120	2.0	5.8	3
Previously characterized global sigma factors and transcriptional factors	RpaB	147	1,600	1.9	7.5	4
	RpaA	12	0	0	9.1 × 10^−10^	2
	RpoD5	52	210	3.1	150,000	6
	RpoD6	3	17	2.3	3,000	2
	NtcA	42	1,570	2.2	27	4
	SigF2	35	2,600	3.6	77,000	5
Previously characterized local TFs	NtcB	3	0	0	9.1 × 10^−10^	1
	IdiB	14	0	0	9.1 × 10^−10^	2
	Fur	8	12	1.3	2.2	2
	Rre1	8	0	0	9.1 × 10^−10^	2
	SufR	8	2.0	0.54	9.6 × 10^−4^	3
	CmpR	8	62	1.8	0.13	2
	Zur	4	2.0	0.73	3.5 × 10^−2^	2
	PerR	18	100	1.88	0.32	3
	SphR	2	1.0	1.1	1.9 × 10^−3^	2
	SmtB	5	0	0	9.1 × 10^−10^	2
	Crp	10	65	2.2	38	4
	KaiC	1	0	0.5	1.9 × 10^−8^	2
	HrcA	185	1,700	2.4	89	4
	GntR	5	8.0	1.1	5.0 × 10^−2^	2
	Pex	58	3,900	4.0	20,000	4
	NblR	0	0	1.5	1.0	1
HI vs. Local ^1^	*p-value*	0.038	0.046	0.035	0.021	0.022

Analysis of centrality metrics across the network revealed distinct patterns. Known global regulators like RpaB showed consistently high centrality scores - ranking in the 92.8^th^ percentile for k-core and 86^th^ percentile for betweenness centrality. Similarly, sigma factors RpoD5 and SigF2 demonstrated elevated centrality metrics. In contrast, local transcriptional regulators like NtcB and IdiB showed markedly lower centrality scores, typically below the 50^th^ percentile, validating the utility of these metrics for identifying high-impact regulators.

### 3.3 Network topology identifies two distinct regulatory subnetworks localizing high centrality transcription factors and coordinating daytime and nighttime metabolic transitions

**Figure 3:**
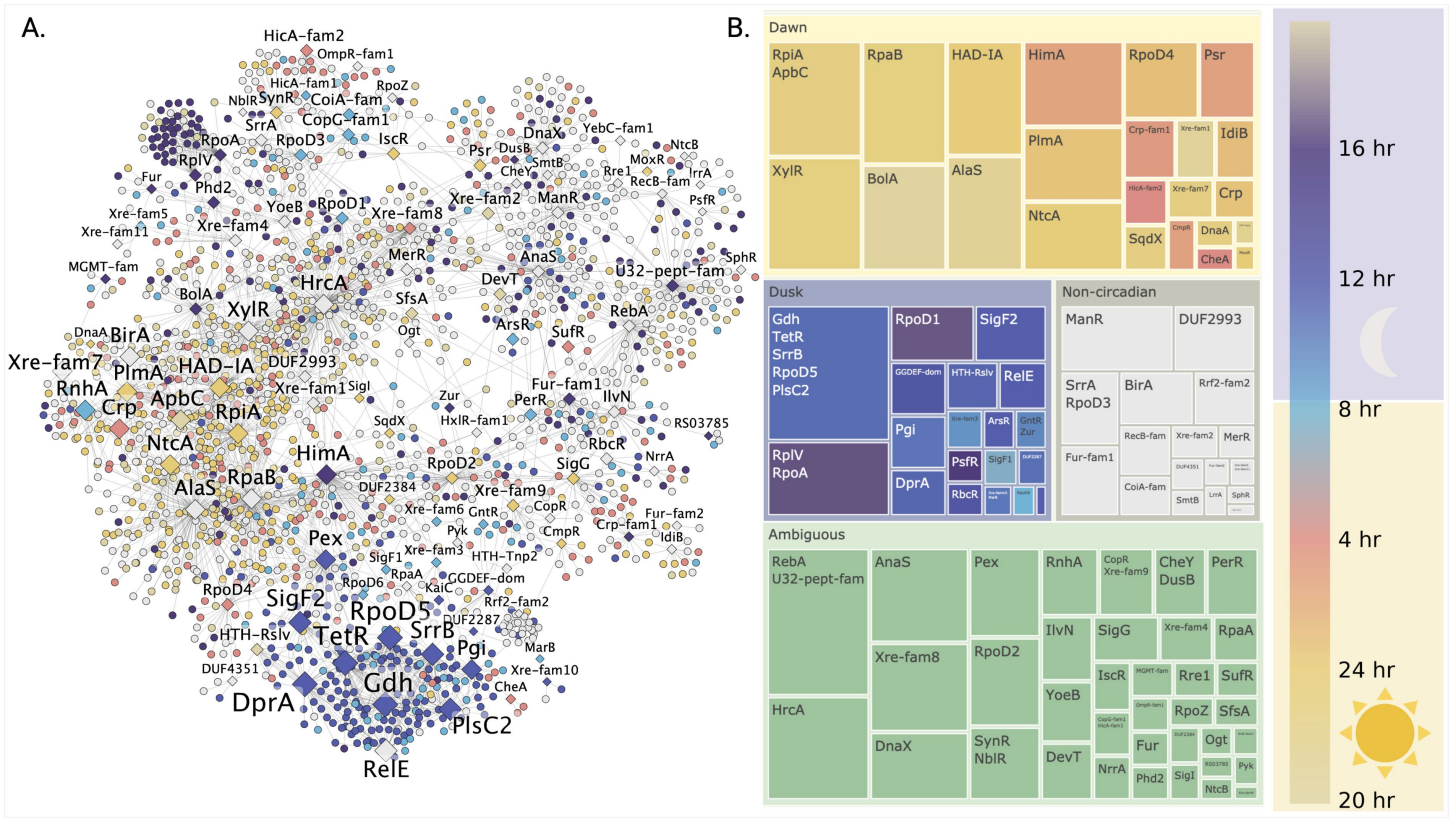
Discovery of temporally organized regulatory networks controlling day-night metabolism in *S. elongatus* PCC 7942. **(A)** Temporal organization of the gene regulatory network across the circadian cycle reveals coordinated waves of gene expression. Genes (circles) and transcription factors (diamonds) are colored by their peak expression times, with TF node size indicating their regulatory influence through k-core centrality. **(B)** Network community analysis uncovers distinct regulatory modules controlling daytime photosynthesis and nighttime metabolism. Each community’s average expression timing (circular mean) reveals how groups of genes are temporally coordinated by their associated transcription factors to enable metabolic transitions.

Having established that network centrality metrics successfully differentiate global from local regulators, we next investigated their organization across the circadian cycle, leveraging *S. elongatus* PCC 7942’s well-characterized circadian rhythms. To understand this temporal organization, we mapped nodes in our inferred gene regulatory network according to their peak expression timing during the circadian cycle. Visualization of transcription factor nodes scaled by k-core values (Figure 3A) revealed two major clusters associated with high centrality regulators - one corresponding to circadian day and another to circadian night. To define regulatory structure within these clusters, we applied the Louvain community detection algorithm (Methods 2.4), labeled communities by their resident transcription factors, and calculated the average peak circadian expression time for genes within each community (Figure 3B). Peak circadian expression was defined according to the results from Vijayan and co-authors (Vijayan et al., 2009) (full circadian dependent gene sets in Supplementary Tables S8, S9).

The largest proportion of regulatory communities lacked clear circadian phase attribution (ambiguous). The most prominent ambiguous communities contained transcriptional regulators RebA and HrcA, with HrcA showing one of the highest network connectivity degrees and known involvement in heat stress response (Saito et al., 2020). The next major group comprised communities with daytime peak expression. Early day transition was marked by communities containing BolA and AlaS regulators. Mid-day communities included global regulator RpaB, sigma factor RpoD4, and nitrogen metabolism regulators NtcA and PlmA. Late day communities contained transcriptional regulators HimA, Psr, and CysR.

Conversely, communities associated with the transition to night were linked to sigma factors SigF1 and RpoD6. Communities with peak expression during mid-circadian night corresponded to a single large community containing three regulators (RpoD5, SrrB, and TetR) and two putative enzymes (Gdh and PlsC2) predicted as potential transcription factors. This community contained nodes with the highest k-core values in the network. Other transcription factors identified in association with circadian night included SigF2 and RbcR, the latter noted as a repressor of Rubisco transcription in *Synechocystis* sp. PCC 6803 (Bolay et al., 2022).

To further characterize the functions associated with these clusters, subgraphs were partitioned by association with circadian day and night. Analysis of transcription factor target overlap revealed that factors with highest degree of overlap also showed highest centrality metrics. Gene set enrichment (Equation 7) analysis against KEGG pathways showed that regulators clustered in the circadian night subgraph (RpaA, RpoD5, and SigF2) were enriched in genes involved in oxidative phosphorylation. In contrast, the circadian day cluster (RpaB, HimA, RpoD4, and NtcA) was enriched in genes associated with photosynthesis and nitrogen metabolism.

### 3.4 Circadian day metabolism reveals hierarchical networks where HimA emerges as a putative DNA architecture regulator working with RpaB and NtcA to coordinate carbon and nitrogen pathways

**Figure 4:**
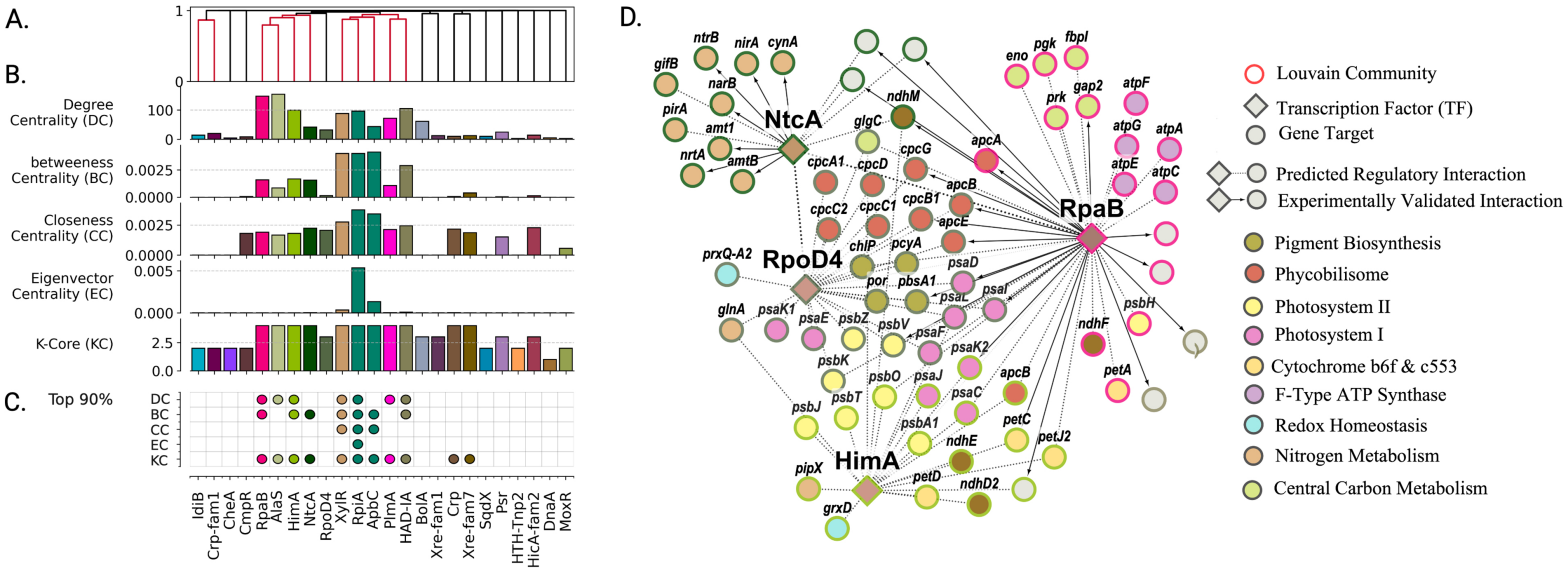
High-centrality transcription factors orchestrate regulatory architecture of daytime carbon fixation and energy metabolism. **(A)** Organization of transcription factors into functional groups based on shared target genes reveals coordinated control of related metabolic processes. Colored clusters indicate TFs sharing >5% gene targets. **(B)** Network centrality analysis identifies TFs with system-wide influence on daytime metabolism. Regulators colored by membership in Louvain community. **(C)** Most influential regulators (top 90th percentile in centrality metrics) emerge as key coordinators of photosynthesis and carbon fixation. **(D)** Functional organization of daytime metabolic regulation showing how RpaB and other high-centrality TFs coordinate photosynthesis, carbon fixation, and energy generation. Node colors indicate metabolic functions; border colors show regulatory communities. Bold arrows highlight experimentally validated interactions captured by network analysis.

After identifying distinct day and night regulatory modules, we performed detailed analysis of the daytime regulatory network to understand how it coordinates photosynthesis, carbon fixation, and nitrogen metabolism. Aligning with its known role as a global regulator (Riediger et al., 2019), RpaB demonstrated exceptionally high centrality metrics within the inferred network (Figures 4B,C), ranking third in degree centrality among all predicted transcription factors. The RpaB node also exhibited high betweenness centrality and k-core values (92.8th and 86th percentiles respectively). Of RpaB’s 97 known targets in the network, 23 were correctly predicted as direct regulatory edges, highlighted by solid edges in Figure 4D. Beyond direct targets, several communities were associated with known RpaB regulation, notably including 9 targets in the high centrality circadian night cluster and 11 targets in a community associated with OmpR family transcription factor SrrA (SYNPCC7942_RS12275) and RpoD3, both known to regulate high light response with RpaB (Seki et al., 2007).

The network community associated with RpaB was significantly enriched for genes involved in central carbon metabolism and energy production (Figure 4D). Specifically, enrichment analysis revealed associations with the Calvin-Benson cycle (map00710, q-value = 5.87 × 10^−3^), F-type ATPase (map00190, q-value = 6.90 × 10^−3^), and components of the photosynthetic electron transport chain (map00195, q-value = 6.87 × 10^−3^). Predicted transcription factors sharing over 5% of gene targets with RpaB’s inferred regulon included HimA, NtcA, and AlaS (Figure 4A). These regulators similarly showed high network centrality, ranking in the top 90th percentile for betweenness centrality and k-core, with AlaS and HimA specifically showing high degree centrality (Figure 4C). The communities associated with these regulators showed distinct functional enrichments - nitrogen metabolism for NtcA (map00910: q-value = 3.04 × 10^−4^) and photosynthesis for HimA (map00195: q-value = 7.67 × 10^−3^). Although RpoD4 did not show notable centrality, its community was significantly enriched in photosynthesis-related genes, including complexes PSI and PSII (map00195: q-value = 1.13 × 10^−11^) as well as antenna proteins (map00196: q-value = 6.74 × 10^−11^).

Together, the RpaB, HimA, and RpoD4 regulons encompass much of the photosynthesis chain and mechanisms associated with cyclic electron flow. Notably, HimA functions as a bacterial nucleoid protein with histone-DNA binding function (Interpro IPR000119) (The UniProt Consortium, 2023) and has been hypothesized to play a role in DNA supercoiling. Previous studies identified HimA as an RpaA target, showing 1.7-fold downregulation in RpaA deletion mutants (Markson et al., 2013). With HimA’s peak expression at circadian night (t = 16 h), and 41/46 circadian genes associated with photosynthesis being negatively correlated with supercoiling (Markson et al., 2013), our network analysis suggests HimA may act as a repressor of the photosynthesis chain through modification of DNA topology.

Finally, nitrogen metabolism was exclusively identified in the circadian day subgraph, where nitrate reduction serves as a major electron sink in PCC 7942 (Grund et al., 2019; Schumann et al., 2023). All nitrogen-related enzymes were associated with NtcA except for the protein regulator PipX (Labella et al., 2016), which was associated with HimA. Through homology analysis with *Synechocystis* PCC6803, we identified two protein-level regulators of nitrogen metabolism, PirA and GifB (García-Domínguez et al., 2000; Bolay et al., 2021), in the NtcA regulon as SYNPCC7942_RS10455 and SYNPCC7942_RS12840, respectively. Functionally enriched gene sets for the circadian day subgraph are provided in Supplementary Table S10.

### 3.5 Circadian night metabolism is hierarchically controlled through RpaA’s indirect orchestration of principal high-centrality regulators TetR, SrrB, and RpoD5 forming single coordinated module

**Figure 5:**
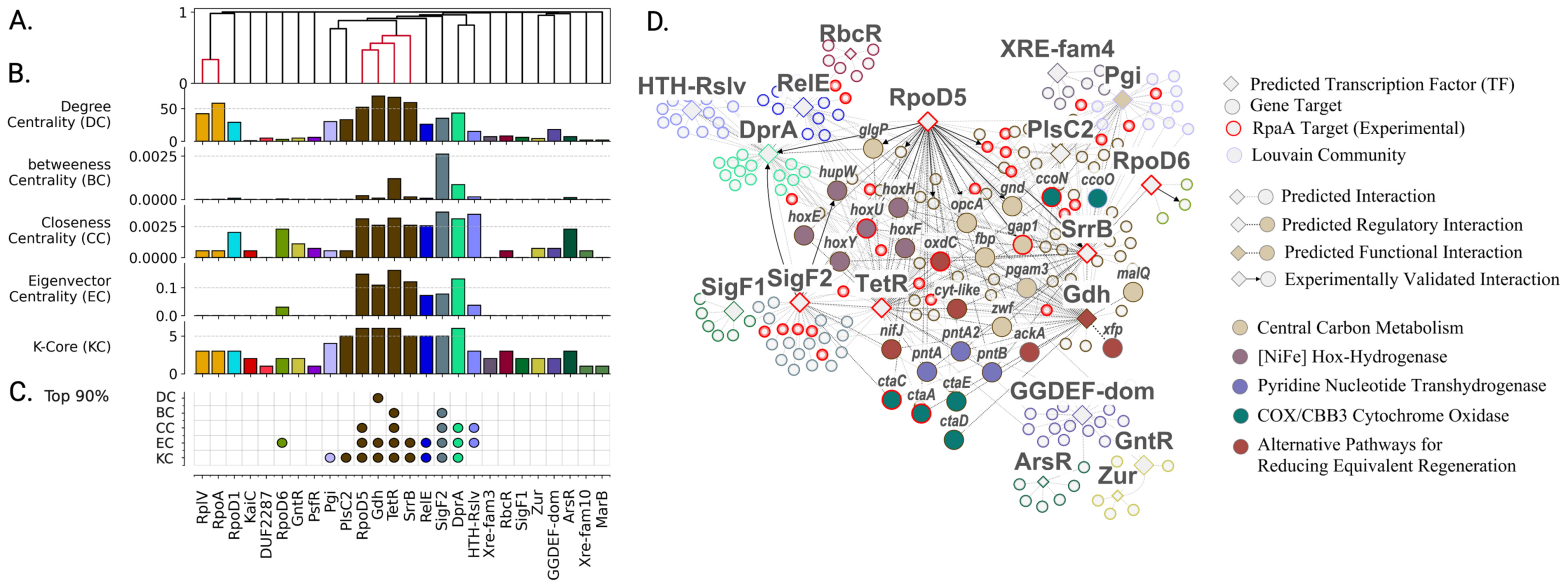
High-centrality transcription factors orchestrate regulatory architecture of nighttime energy generation and redox metabolism. **(A)** Organization of transcription factors into functional groups based on shared target genes reveals tightly coordinated control of nighttime processes. Colored clusters indicate TFs sharing >30% gene targets. **(B)** Network centrality analysis identifies TFs with system-wide influence on nighttime metabolism. Regulators colored by membership in Louvain community. **(C)** Most influential regulators (top 90th percentile in centrality metrics) emerge as key coordinators of glycogen mobilization and reducing power generation. **(D)** Functional organization of nighttime metabolic regulation showing how RpaA-controlled TFs coordinate energy generation and redox balance. Node colors indicate regulatory communities; red borders highlight experimentally validated RpaA targets. Enriched protein complexes and metabolic functions are labeled.

While daytime metabolism showed distributed control through multiple regulatory communities, our analysis revealed a surprisingly different organization of nighttime metabolism with implications for understanding temporal control of cellular resources. The network centrality metrics did not fully capture RpaA’s important regulatory role. Of RpaA’s 102 reported targets, 74 were represented in the predicted gene regulatory network. However, with only 12 predicted edges (5 correctly predicted, including circadian oscillator protein KaiB), RpaA ranked in lower percentiles for all centrality measures: degree (47.3%), k-core (27.5%), betweenness (15.3%), eigenvector (13.1%), and closeness (13.1%). Most known RpaA targets were instead captured in communities associated with its downstream transcription factors identified in ChIP-seq studies. Most notably, the gene set community associated with regulators TetR, SrrB, and RpoD5 contained 20 known RpaA targets (Figure 5D), with additional targets appearing in communities of SigF2 (7 genes) and HimA (3 genes).

The gene regulatory network captured RpaA’s regulatory influence through its downstream regulators, including sigma factors RpoD5, SigF2, RpoD6, and transcription factors SrrB and TetR - all showing peak expression at night and high network centrality (Figures 5B,C). These regulators demonstrated substantial target overlap, with SrrB, TetR, and RpoD5 sharing 30% of gene targets (Figure 5A). SigF2 ranked among the top nodes across all metrics (>95th percentile). RpoD5 showed similarly high values in closeness centrality (93.2%), eigenvector centrality (99.1%), and k-core (98.6%). While RpoD6 had only three regulatory edges, its connection to high-impact targets placed its eigenvector centrality in the 92.8th percentile. Transcription factors SrrB and TetR demonstrated exceptionally high centrality values (>80th percentile across all metrics), with TetR showing the highest eigenvector centrality in the network and SrrB ranking fourth (97.3%).

Unlike the multiple gene set communities associated with peak expression during circadian day, only a single gene set community emerged during the transition to circadian night. This community appeared to be tightly coregulated, evidenced by substantial transcription factor overlap (Figure 5A) and high centrality scores (Figure 5C), particularly in metrics indicating closeness to other central regulators. Gene set enrichment analysis revealed significant associations with energy production and conversion, and carbohydrate metabolism (Figure 5C). Specifically, genes in this community were enriched in KEGG pathways for oxidative phosphorylation (map00190, q-value = 4.5 × 10^−4^) and pentose phosphate pathway (map00030, q-value = 4.5 × 10^−3^).

The presence of a single, tightly coregulated, functionally coherent gene set associated with the transition to circadian night in PCC 7942 reflects a tightly regulated metabolic shift. This shift is primarily driven by the necessity to regenerate reducing power and ATP in the absence of photosynthesis and involves several key processes. Glycogen breakdown, catalyzed by GlgP and MalQ, fuels NADPH generation through the oxidative pentose phosphate pathway (enzymes *zwf*, *opcA*, and *gnd*). ATP synthesis is maintained through respiration-driven proton gradients associated with cytochromes CtaACDE and CcoNO. Alternative pathways for reducing power generation and Calvin-Benson cycle intermediate recycling are also identified in the cluster: PntAB for NADH to NADPH conversion; the phosphoketolase (Xfp) (SYNPCC7942_RS10545) acetate kinase (AckA) pathway involved in ATP sensing and conversion of Calvin-Benson cycle intermediates (Lu et al., 2023); glucose-1-dehydrogenase (Gdh) involved in direct glucose oxidation; and oxalate oxidation via oxalate decarboxylase (OxdC) and a predicted formate dehydrogenase (SYNPCC7942_RS12130) (Schwarz et al., 2011). Finally, NifJ and the HOX bidirectional NiFe dehydrogenase *hoxEFUH* were identified as potentially coupling ferredoxin oxidation to hydrogen reduction, as described in *Synechocystis* PCC 6803 under anaerobic dark conditions (Maus et al., 2016; Khanna and Lindblad, 2015). Interestingly, succinate dehydrogenase, the primary electron donator attributed to plastoquinone reduction (Liu et al., 2012; Lee et al., 2007), was not identified in the circadian night cluster. Functionally enriched gene sets for the circadian night subgraph are provided in Supplementary Table S11.

## 4 Discussion

### 4.1 Strengths of GRN inference: network topology analysis reveals potential key regulatory elements despite limited direct TF-gene prediction accuracy

Network analysis of gene regulation offers a powerful alternative to traditional approaches focused on individual interactions. Our study of circadian regulation in PCC 7942 demonstrates how network-level analysis can reveal biologically meaningful insights even when direct transcription factor-gene predictions show limited accuracy. By examining the emergent properties of the regulatory network - its topology, community structure, and centrality patterns - we identified regulatory principles that align with and extend current knowledge of cyanobacterial metabolism. Through network centrality analysis, we discovered three high-confidence transcription factors (HimA, TetR, and SrrB) as promising candidates for experimental characterization. These factors show distinct patterns of circadian phase association and appear to coordinate key metabolic transitions between day and night, with direct implications for optimizing photosynthetic bioproduction. Table 2 summarizes key characteristics of these regulators, their temporal regulation, and predicted functions based on network analysis.

**Table 2:** Previously uncharacterized transcription factors identified as key circadian regulators through network analysis based on their high centrality positions.

Circadian phase	Name	Gene ID	Description	Ref.
Day	HimA	SYNPCC7942_RS11420	Histone-like protein with putative role in DNA supercoiling. Peaked expression at night, high network centrality and connections to photosynthesis-related suggests role in repression of day-phase processes through DNA topology	Vijayan et al. (2009) and Markson et al. (2013)
Night	SrrB	SYNPCC7942_RS02830	Response regulator interacting with histidine kinase. Its high network centrality suggests unexplored broader regulatory influence over night-phase metabolism	Sierro et al. (2008)
Night	TetR	SYNPCC7942_RS03075	Regulator previously linked to Kai-dependent dark-induced genes. Network analysis suggests its broader regulatory role in nighttime metabolism	Welkie et al. (2019) and Colclough et al. (2019)

### 4.2 Challenges of GRN inference: current limitations in TF prediction and network construction highlight areas for methodological advancement

Our analysis revealed two major challenges in GRN inference. The first challenge involves the lack of consensus in transcription factor identification across different computational prediction methods. Despite using three complementary computational approaches (P2TF, ENTRAF, and DeepTFactor), we found substantial variation in TF predictions, highlighting the difficulty in definitively identifying transcriptional regulators. While some predicted TFs might have moonlighting functions beyond their known metabolic roles, their identification should be interpreted cautiously and requires experimental validation (Supplementary Table S12). These challenges underscore the importance of integrating computational predictions with experimental data and the need for continued refinement of TF prediction methods in cyanobacteria (full list of predicted TFs in Supplementary Table S5).

The second, more significant challenge lies in accurately predicting direct TF-gene regulatory interactions. Despite achieving network-level insights that align with known biology, our ability to predict individual regulatory connections remains limited, with precision and recall values similar to those reported in other studies of bacterial networks. This moderate accuracy in predicting direct interactions likely stems from both the TF prediction uncertainties described above and the inherent complexity and hierarchical nature of transcriptional regulation, including effects of DNA topology, protein–protein interactions, and various post-transcriptional mechanisms. The inclusion of potential false positive TFs in our inclusive approach may have contributed to spurious regulatory connection predictions, further reducing prediction accuracy. These additional regulatory layers, discussed further in section 4.4, suggest that future improvements in prediction accuracy may require integration of multiple data types beyond gene expression.

### 4.3 Elucidating regulation of day-night transitions: network analysis reveals complex coordination of carbon, nitrogen and redox metabolism with implications for bioproduction

Our network analysis reveals a sophisticated regulatory architecture that orchestrates metabolic transitions between day and night phases in *S. elongatus* PCC 7942. The significance of understanding this temporal control is highlighted by recent findings showing that circadian regulation can modulate heterologous product yields up to three-fold, when comparing cultures recently transitioned to constant light and having same CO₂ supply (Gilliam et al., 2025). By mapping regulatory networks, we uncovered potential key control points for carbon allocation, nitrogen assimilation, and redox balance across diurnal cycles – insights that can be directly applicable to metabolic engineering strategies.

During subjective day, the network demonstrates coordinated control of photosynthetic and carbon fixation machinery through global regulator RpaB and potential co-regulators HimA and RpoD4 (Figure 4D). These regulators activate components beneficial for bioproduction, including PSII and phycobilisome complexes (*psb*, *cpc*), ATP synthesis machinery, and carbon fixation pathways (*gap2*, *prk*, *eno*, *fbpI*, *pgk*). The same regulators control electron transport chain components, including genes encoding Cyt c6 and Cyt c553, and NADH dehydrogenase genes. However, coordinated action of RpaB and RpoD4 could also activate competing sinks for carbon and reducing power (Gilliam et al., 2025), including glycogen biosynthesis (through activation of glucose-1-phosphate adenylyltransferase, *glgC*). An additional competing sink for reducing power is nitrate reduction, regulated by NtcA.

During subjective night, our analysis reveals activation of an intricate regulatory program that mobilizes stored carbon while maintaining cellular redox balance. As demonstrated in our recent study (Gilliam et al., 2025), enhancing product yield might be more effectively achieved through glycogen as an intermediate product, rather than disrupting its synthesis, which can result in severe metabolic imbalances, reduced efficiency of light capture and carbon fixation, and elevated sensitivity to stresses (Guerra et al., 2013; Cantrell et al., 2023; Hickman et al., 2013; Miao et al., 2003; Gründel et al., 2012). Our network analysis identified a complex regulatory network rewiring carbon and redox machinery, including enzymes related to glycogen degradation (GlgP, MalQ), oxidative pentose phosphate pathway (*zwf*, *opcA*, *gnd*) generating NADPH, and respiratory electron transport chain cytochromes (CytC and Cyt aa3). The network also identifies activation of alternative pathways for reducing power generation, including the HOX bidirectional NiFe hydrogenase (complex encoded by *hoxEFUH*) and glucose-1-dehydrogenase (Gdh).

In the network of regulators activating night metabolism, our analysis identified HimA, TetR, and SrrB as high-centrality transcription factors and potential key orchestrators. These regulators show extensive connections to both carbon metabolism and redox balance pathways, suggesting they may play central roles in coordinating the metabolic state transitions that enable nighttime productivity. This improved understanding of the regulatory networks governing day-night metabolic transitions may provide valuable insights for metabolic engineering strategies aimed at enhancing temporal control of bioproduction in cyanobacterial systems. Future studies can build upon this regulatory framework to develop more sophisticated approaches for temporal optimization of metabolic pathways and resource allocation.

### 4.4 Future perspectives: integration of multi-omics data and advanced computational approaches will enhance understanding of cellular regulation

Our study demonstrates both the potential and current limitations of machine learning-based GRN inference in elucidating the regulatory landscape of *S. elongatus* PCC 7942. While our network-level analysis successfully revealed biologically meaningful patterns, several key challenges remain to be addressed. These include accurate prediction of direct regulatory interactions, identification of true transcription factors, and capturing complex regulatory mechanisms beyond transcriptional control. We envision three complementary approaches to address these challenges.

#### 4.4.1 Integration of multi-omics data

A critical next step is the integration of transcriptomics with proteomics, metabolomics, and epigenomics data to provide a more comprehensive view of cellular regulation. Multiple regulatory mechanisms are known in cyanobacteria, including DNA topological compaction (Vijayan et al., 2009; Woelfle et al., 2007), DNA methylation (Gärtner et al., 2019), non-coding RNA regulation (Brenes-Álvarez et al., 2021), ribosome allocation (Karlsen et al., 2018), ribonuclease mRNA degradation (Hoffmann et al., 2021), and protein phosphorylation and cysteine modification (Cheng et al., 2024; Jimbo et al., 2018; Horiuchi et al., 2010; Nakamura and Hihara, 2006; Cheung et al., 2024). As new methods for measuring global cell activity continue to develop, integrating these multiple regulatory signals (Argelaguet et al., 2018) may provide additional constraints for network predictions.

#### 4.4.2 Advanced computational approaches

Novel ML and AI architectures show promise for integrating multiple data types into systems models. Successful approaches include independent components analysis (ICA) (Patel et al., 2023), graph neural networks (GNN) (Cao and Gao, 2022), multi-omics factor analysis (MOFA) (Argelaguet et al., 2018), and multi-omics analysis based on physics-informed machine learning (Johnson et al., 2024). These methods offer ways to leverage diverse data types while accounting for their distinct characteristics and relationships.

#### 4.4.3 Integration with metabolic models

Combining transcriptional systems models with metabolic models could provide a more comprehensive understanding of cellular behavior (Chen et al., 2024; Domenzain et al., 2022; Shin et al., 2024). This integration would bridge the gap between regulatory networks and metabolic fluxes, potentially improving our ability to predict and engineer cellular phenotypes. Such integrated models could enhance our understanding of how regulatory changes impact metabolic outcomes, particularly important for biotechnology applications.

These future directions aim to address current limitations while expanding the utility of GRN inference in understanding and manipulating cyanobacterial metabolism. Success in these areas could significantly advance both fundamental knowledge and biotechnology applications.

## 5 Conclusion

This study addresses a fundamental challenge in systems biology: extracting actionable insights from complex gene expression datasets despite limitations in predicting individual regulatory interactions. By integrating machine learning with network topology analysis, we demonstrate how network-level features can reveal biologically meaningful patterns even when gene regulatory network shows moderate accuracy in predicting individual TF-gene interactions - a common challenge in the field. Through analysis of network centrality metrics and community structure rather than individual edge predictions, we identified three previously understudied transcription factors with potentially significant regulatory roles: HimA as a putative DNA architecture regulator orchestrating photosynthetic gene expression through topological control, and TetR and SrrB as key coordinators of nighttime metabolism.

The network analysis revealed distinct regulatory modules associated with circadian day and night phases, elucidating how global and local transcriptional regulators work in concert to coordinate complex metabolic transitions. During the day phase, we found hierarchical regulation of photosynthesis and carbon fixation through RpaB, HimA, and RpoD4, while nighttime metabolism showed tight coordination through a single regulatory module centered around RpaA’s indirect control through TetR, SrrB, and RpoD5. Understanding this temporal organization of metabolism is particularly relevant for biotechnology applications, where coordinated control of carbon fixation and energy generation directly impacts photosynthetic bioproduction efficiency.

Our findings demonstrate that network-level analysis can reveal biologically meaningful insights even when direct regulatory interaction predictions are limited. This approach has broad implications beyond cyanobacterial research, offering a framework for analyzing complex regulatory networks in photosynthetic and heterotrophic organisms where experimental validation of individual interactions remains challenging. The methodologies presented here can advance our understanding of metabolic regulation across diverse microbial systems, potentially facilitating the development of more sophisticated metabolic engineering strategies for enhanced carbon fixation and sustainable bioproduction.

## Data Availability

Publicly available datasets analyzed in this study can be found in online repositories. The names of the repository/repositories and accession numbers can be found in the article/Supplementary material.

## References

[ref1] Abdulrehman D. Monteiro P. T. Teixeira M. C. Mira N. P. Lourenço A. B. Dos Santos S. C.. (2011). Yeastract: providing a programmatic access to curated transcriptional regulatory associations in *Saccharomyces cerevisiae* through a web services interface. Nucleic Acids Res. 39, D136–D140. doi: 10.1093/nar/gkq964, PMID: 20972212 PMC3013800

[ref2] Abramson B. W. Kachel B. Kramer D. M. Ducat D. C. (2016). Increased photochemical efficiency in Cyanobacteria via an engineered sucrose sink. Plant Cell Physiol. 57, 2451–2460. doi: 10.1093/pcp/pcw169, PMID: 27742883

[ref3] Altaf-Ul-Amine M. Nishikata K. Korna T. Miyasato T. Shinbo Y. Arifuzzaman M.. (2003). Prediction of protein functions based on K-cores of protein-protein interaction networks and amino acid sequences. Genome Inform. 14, 498–499. doi: 10.11234/gi1990.14.498

[ref4] Andrew S. (2010). *Fastqc. A quality control tool for high throughput sequence data*.

[ref5] Argelaguet R. Velten B. Arnol D. Dietrich S. Zenz T. Marioni J. C.. (2018). Multi-omics factor analysis-a framework for unsupervised integration of multi-omics data sets. Mol. Syst. Biol. 14:e8124. doi: 10.15252/msb.20178124, PMID: 29925568 PMC6010767

[ref6] Barrett T. Wilhite S. E. Ledoux P. Evangelista C. Kim I. F. Tomashevsky M.. (2013). Ncbi geo: archive for functional genomics data sets—update. Nucleic Acids Res. 41, D991–D995. doi: 10.1093/nar/gks1193, PMID: 23193258 PMC3531084

[ref7] Batagelj V. Zaversnik M. (2003). *An O (m) algorithm for cores decomposition of networks*. Cornell University.

[ref8] Bhardwaj N. Yan K.-K. Gerstein M. B. (2010). Analysis of diverse regulatory networks in a hierarchical context shows consistent tendencies for collaboration in the middle levels. Proc. Natl. Acad. Sci. 107, 6841–6846. doi: 10.1073/pnas.0910867107, PMID: 20351254 PMC2872381

[ref9] Bolay P. Rozbeh R. Muro-Pastor M. I. Timm S. Hagemann M. Florencio F. J.. (2021). The novel P(ii)-interacting protein PirA controls flux into the cyanobacterial ornithine-Ammonia cycle. MBio 12:e00229. doi: 10.1128/mBio.00229-21 33758091 PMC8092223

[ref10] Bolay P. Schlüter S. Grimm S. Riediger M. Hess W. R. Klähn S. (2022). The transcriptional regulator RbcR controls ribulose-1,5-bisphosphate carboxylase/oxygenase (RuBisco) genes in the cyanobacterium *Synechocystis* sp. Pcc 6803. New Phytol. 235, 432–445. doi: 10.1111/nph.18139, PMID: 35377491

[ref11] Brenes-Álvarez M. Olmedo-Verd E. Vioque A. Muro-Pastor A. M. (2021). A nitrogen stress-inducible small Rna regulates Co_2_ fixation in Nostoc. Plant Physiol. 187, 787–798. doi: 10.1093/plphys/kiab309, PMID: 34608966 PMC8491059

[ref12] Cantrell M. Cano M. Sebesta J. Paddock T. Xiong W. Chou K. J.. (2023). Manipulation of glycogen and sucrose synthesis increases photosynthetic productivity in cyanobacteria. Front. Microbiol. 14:274. doi: 10.3389/fmicb.2023.1124274, PMID: 37275163 PMC10233058

[ref13] Cao Z.-J. Gao G. (2022). Multi-omics single-cell data integration and regulatory inference with graph-linked embedding. Nat. Biotechnol. 40, 1458–1466. doi: 10.1038/s41587-022-01284-4 35501393 PMC9546775

[ref14] Chen Y. Gustafsson J. Tafur Rangel A. Anton M. Domenzain I. Kittikunapong C.. (2024). Reconstruction, simulation and analysis of enzyme-constrained metabolic models using gecko toolbox 3.0. Nat. Protoc. 19, 629–667. doi: 10.1038/s41596-023-00931-7, PMID: 38238583

[ref15] Cheng C. Lu D. Sun H. Zhang K. Yin L. Luan G.. (2024). Structural insight into the functional regulation of elongation factor Tu by reactive oxygen species in *Synechococcus elongatus* Pcc 7942. Int. J. Biol. Macromol. 277:133632. doi: 10.1016/j.ijbiomac.2024.133632, PMID: 38971279

[ref16] Cheung M. S. Mejia-Rodriguez D. Kim H. Sadler N. Li X. Bohutskyi P.. (2024). Ptm-psi: a Python package to facilitate the computational investigation of post-translational modification on protein structures and their impacts on dynamics and functions. Biophys. J. 123, 354a–355a. doi: 10.1016/j.bpj.2023.11.2147 PMC10659954 37902126

[ref17] Choi K. R. Jang W. D. Yang D. Cho J. S. Park D. Lee S. Y. (2019). Systems metabolic engineering strategies: integrating systems and synthetic biology with metabolic engineering. Trends Biotechnol. 37, 817–837. doi: 10.1016/j.tibtech.2019.01.003, PMID: 30737009

[ref18] Colclough A. L. Scadden J. Blair J. M. A. (2019). TetR-family transcription factors in gram-negative bacteria: conservation, variation and implications for efflux-mediated antimicrobial resistance. BMC Genomics 20:731. doi: 10.1186/s12864-019-6075-5, PMID: 31606035 PMC6790063

[ref19] Del Rio G. Koschützki D. Coello G. (2009). How to identify essential genes from molecular networks? BMC Syst. Biol. 3:102. doi: 10.1186/1752-0509-3-102 19822021 PMC2765966

[ref20] Domenzain I. Sánchez B. Anton M. Kerkhoven E. J. Millán-Oropeza A. Henry C.. (2022). Reconstruction of a catalogue of genome-scale metabolic models with enzymatic constraints using gecko 2.0. Nat. Commun. 13:3766. doi: 10.1038/s41467-022-31421-1 35773252 PMC9246944

[ref21] Escorcia-Rodríguez J. M. Gaytan-Nuñez E. Hernandez-Benitez E. M. Zorro-Aranda A. Tello-Palencia M. A. Freyre-González J. A. (2023). Improving gene regulatory network inference and assessment: The importance of using network structure. Front. Genet. 14:382. doi: 10.3389/fgene.2023.1143382, PMID: 36926589 PMC10012345

[ref22] Fang X. Sastry A. Mih N. Kim D. Tan J. Yurkovich J. T.. (2017). Global transcriptional regulatory network for *Escherichia coli* robustly connects gene expression to transcription factor activities. Proc. Natl. Acad. Sci. 114, 10286–10291. doi: 10.1073/pnas.1702581114, PMID: 28874552 PMC5617254

[ref23] Fleming K. (2017). *A clock-phased sigma factor Cascade is required for global circadian transcriptional rhythms in Cyanobacteria*. Doctor of Philosophy in Biology, Harvard University.

[ref24] Fleming K. E. O’shea E. K. (2018). An RpaA-dependent sigma factor Cascade sets the timing of circadian transcriptional rhythms in *Synechococcus elongatus*. Cell Rep. 25, 2937–2945.e3. doi: 10.1016/j.celrep.2018.11.049, PMID: 30540929

[ref25] Freeman L. C. (1977). A set of measures of centrality based on Betweenness. Sociometry 40, 35–41. doi: 10.2307/3033543

[ref26] Freeman L. C. (1978). Centrality in social networks conceptual clarification. Soc. Networks 1, 215–239. doi: 10.1016/0378-8733(78)90021-7

[ref27] García-Domínguez M. Reyes J. C. Florencio F. J. (2000). NtcA represses transcription of gifA and gifB, genes that encode inhibitors of glutamine synthetase type I from Synechocystis sp. Pcc 6803. Mol. Microbiol. 35, 1192–1201. doi: 10.1046/j.1365-2958.2000.01789.x, PMID: 10712699

[ref28] Gärtner K. Klähn S. Watanabe S. Mikkat S. Scholz I. Hess W. R.. (2019). Cytosine N4-methylation via M.Ssp6803ii is involved in the regulation of transcription, fine-tuning of Dna replication and Dna repair in the cyanobacterium *Synechocystis* sp. Pcc 6803. Front. Microbiol. 10:1233. doi: 10.3389/fmicb.2019.01233 31231331 PMC6560206

[ref29] Ghamrawi N. Mccallum A. (2005). *Collective multi-label classification*. Proceedings of the 14th Acm international conference on information and knowledge management. Bremen, Germany: Association for Computing Machinery.

[ref30] Gilliam A. Sadler N. C. Li X. Garcia M. Johnson J. Veličković M.. (2025). Cyanobacterial circadian regulation enhances bioproduction under subjective nighttime through rewiring of carbon partitioning dynamics, redox balance orchestration, and cell cycle modulation. Microb. Cell Factories 24:56. doi: 10.1186/s12934-025-02665-5 PMC11889915 40055679

[ref31] Greenfield A. Madar A. Ostrer H. Bonneau R. (2010). Dream4: combining genetic and dynamic information to identify biological networks and dynamical models. PLoS One 5:e13397. doi: 10.1371/journal.pone.0013397, PMID: 21049040 PMC2963605

[ref32] Grund M. Jakob T. Wilhelm C. Bühler B. Schmid A. (2019). Electron balancing under different sink conditions reveals positive effects on photon efficiency and metabolic activity of *Synechocystis* sp. Pcc 6803. Biotechnol. Biofuels 12:43. doi: 10.1186/s13068-019-1378-y, PMID: 30858880 PMC6391784

[ref33] Gründel M. Scheunemann R. Lockau W. Zilliges Y. (2012). Impaired glycogen synthesis causes metabolic overflow reactions and affects stress responses in the cyanobacterium *Synechocystis* sp. Pcc 6803. Microbiology 158, 3032–3043. doi: 10.1099/mic.0.062950-0, PMID: 23038809

[ref34] Guerra L. T. Xu Y. Bennette N. Mcneely K. Bryant D. A. Dismukes G. C. (2013). Natural osmolytes are much less effective substrates than glycogen for catabolic energy production in the marine cyanobacterium *Synechococcus* sp. strain Pcc 7002. J. Biotechnol. 166, 65–75. doi: 10.1016/j.jbiotec.2013.04.005, PMID: 23608552

[ref35] Gutu A. O’shea E. K. (2013). Two antagonistic clock-regulated histidine kinases time the activation of circadian gene expression. Mol. Cell 50, 288–294. doi: 10.1016/j.molcel.2013.02.022, PMID: 23541768 PMC3674810

[ref36] Hagberg A. Swart P. Chult S. D. (2008). *Exploring network structure, dynamics, and function using NetworkX*. Los Alamos National Lab. (Lanl), Los Alamos, Nm (United States).

[ref37] Hanaoka M. Takai N. Hosokawa N. Fujiwara M. Akimoto Y. Kobori N.. (2012). RpaB, another response regulator operating circadian clock-dependent transcriptional regulation in *Synechococcus elongatus* Pcc 7942. J. Biol. Chem. 287, 26321–26327. doi: 10.1074/jbc.M111.338251, PMID: 22665493 PMC3406716

[ref38] Häusler S. (2024). Correlations reveal the hierarchical organization of biological networks with latent variables. Commun. Biol. 7:678. doi: 10.1038/s42003-024-06342-y, PMID: 38831002 PMC11148204

[ref39] Hickman J. W. Kotovic K. M. Miller C. Warrener P. Kaiser B. Jurista T.. (2013). Glycogen synthesis is a required component of the nitrogen stress response in *Synechococcus elongatus* Pcc 7942. Algal Res. 2, 98–106. doi: 10.1016/j.algal.2013.01.008

[ref40] Hoffmann U. A. Heyl F. Rogh S. N. Wallner T. Backofen R. Hess W. R.. (2021). Transcriptome-wide in vivo mapping of cleavage sites for the compact cyanobacterial ribonuclease E reveals insights into its function and substrate recognition. Nucleic Acids Res. 49, 13075–13091. doi: 10.1093/nar/gkab1161, PMID: 34871439 PMC8682795

[ref41] Horiuchi M. Nakamura K. Kojima K. Nishiyama Y. Hatakeyama W. Hisabori T.. (2010). The PedR transcriptional regulator interacts with thioredoxin to connect photosynthesis with gene expression in cyanobacteria. Biochem. J. 431, 135–140. doi: 10.1042/BJ20100789 20662766

[ref42] Hudson E. P. (2023). The Calvin Benson cycle in bacteria: new insights from systems biology. Semin. Cell Dev. Biol. 155, 71–83. doi: 10.1016/j.semcdb.2023.03.007, PMID: 37002131

[ref43] Huerta A. M. Salgado H. Thieffry D. Collado-Vides J. (1998). Regulondb: a database on transcriptional regulation in *Escherichia coli*. Nucleic Acids Res. 26, 55–59. doi: 10.1093/nar/26.1.55, PMID: 9399800 PMC147189

[ref44] Huynh-Thu V. A. Irrthum A. Wehenkel L. Geurts P. (2010). Inferring regulatory networks from expression data using tree-based methods. PLoS One 5:e12776. doi: 10.1371/journal.pone.0012776, PMID: 20927193 PMC2946910

[ref45] Iglesias-Martinez L. F. De Kegel B. Kolch W. (2021). Kboost: a new method to infer gene regulatory networks from gene expression data. Sci. Rep. 11:15461. doi: 10.1038/s41598-021-94919-6, PMID: 34326402 PMC8322418

[ref46] Ishiura M. Kutsuna S. Aoki S. Iwasaki H. Andersson C. R. Tanabe A.. (1998). Expression of a gene cluster kaiabc as a circadian feedback process in cyanobacteria. Science 281, 1519–1523. doi: 10.1126/science.281.5382.1519, PMID: 9727980

[ref47] Jimbo H. Yutthanasirikul R. Nagano T. Hisabori T. Hihara Y. Nishiyama Y. (2018). Oxidation of translation factor Ef-Tu inhibits the repair of photosystem ii. Plant Physiol. 176, 2691–2699. doi: 10.1104/pp.18.00037, PMID: 29439212 PMC5884602

[ref48] Johnson C. G. M. Johnson Z. Mackey L. S. Li X. Sadler N. C. Zhang T.. (2024). *Transcriptome and redox proteome reveal temporal scales of carbon metabolism regulation in model Cyanobacteria under light disturbance*. arXiv, 2410.09346.

[ref49] Jothi R. Balaji S. Wuster A. Grochow J. A. Gsponer J. Przytycka T. M.. (2009). Genomic analysis reveals a tight link between transcription factor dynamics and regulatory network architecture. Mol. Syst. Biol. 5:294. doi: 10.1038/msb.2009.52, PMID: 19690563 PMC2736650

[ref50] Kanehisa M. Goto S. (2000). Kegg: Kyoto encyclopedia of genes and genomes. Nucleic Acids Res. 28, 27–30. doi: 10.1093/nar/28.1.27, PMID: 10592173 PMC102409

[ref51] Karlsen J. Asplund-Samuelsson J. Thomas Q. Jahn M. Hudson Elton P. (2018). Ribosome profiling of Synechocystis reveals altered ribosome allocation at carbon starvation. mSystems 3:18. doi: 10.1128/msystems.00126-18

[ref52] Karp P. D. Billington R. Caspi R. Fulcher C. A. Latendresse M. Kothari A.. (2019). The BioCyc collection of microbial genomes and metabolic pathways. Brief. Bioinform. 20, 1085–1093. doi: 10.1093/bib/bbx085, PMID: 29447345 PMC6781571

[ref53] Kato H. Chibazakura T. Yoshikawa H. (2008). NblR is a novel one-component response regulator in the cyanobacterium *Synechococcus elongatus* Pcc 7942. Biosci. Biotechnol. Biochem. 72, 1072–1079. doi: 10.1271/bbb.70816, PMID: 18391440

[ref54] Katz K. Shutov O. Lapoint R. Kimelman M. Brister J. R. O’sullivan C. (2022). The sequence read archive: a decade more of explosive growth. Nucleic Acids Res. 50, D387–D390. doi: 10.1093/nar/gkab1053, PMID: 34850094 PMC8728234

[ref55] Khanna N. Lindblad P. (2015). Cyanobacterial hydrogenases and hydrogen metabolism revisited: recent progress and future prospects. Int. J. Mol. Sci. 16, 10537–10561. doi: 10.3390/ijms160510537, PMID: 26006225 PMC4463661

[ref56] Kim G. B. Gao Y. Palsson B. O. Lee S. Y. (2021). Deeptfactor: a deep learning-based tool for the prediction of transcription factors. Proc. Natl. Acad. Sci. 118:e2021171118. doi: 10.1073/pnas.2021171118, PMID: 33372147 PMC7812831

[ref57] Ko Y.-S. Kim J. W. Lee J. A. Han T. Kim G. B. Park J. E.. (2020). Tools and strategies of systems metabolic engineering for the development of microbial cell factories for chemical production. Chem. Soc. Rev. 49, 4615–4636. doi: 10.1039/D0CS00155D, PMID: 32567619

[ref58] Kong Y.-X. Shi G.-Y. Wu R.-J. Zhang Y.-C. (2019). K-core: theories and applications. Phys. Rep. 832, 1–32. doi: 10.1016/j.physrep.2019.10.004

[ref59] Koschutzki D. Schreiber F. (2008). Centrality analysis methods for biological networks and their application to gene regulatory networks. Gene Regul. Syst. Biol. 2, 193–201. doi: 10.4137/grsb.s702, PMID: 19787083 PMC2733090

[ref60] Kutsuna S. Kondo T. Ikegami H. Uzumaki T. Katayama M. Ishiura M. (2007). The circadian clock-related gene pex regulates a negative cis element in the kaiA promoter region. J. Bacteriol. 189, 7690–7696. doi: 10.1128/JB.00835-07, PMID: 17704219 PMC2168723

[ref61] Labella J. I. Obrebska A. Espinosa J. Salinas P. Forcada-Nadal A. Tremino L.. (2016). Expanding the cyanobacterial nitrogen regulatory network: The GntR-like regulator PlmA interacts with the Pii-PipX complex. Front. Microbiol. 7:1677. doi: 10.3389/fmicb.2016.01677, PMID: 27840625 PMC5083789

[ref62] Ledesma L. Hernandez-Guerrero R. Perez-Rueda E. (2022). Prediction of Dna-binding transcription factors in Bacteria and Archaea genomes. Methods Mol Biol 2516, 103–112. doi: 10.1007/978-1-0716-2413-5_7, PMID: 35922624

[ref63] Lee S. Y. Kim H. U. (2015). Systems strategies for developing industrial microbial strains. Nat. Biotechnol. 33, 1061–1072. doi: 10.1038/nbt.3365, PMID: 26448090

[ref64] Lee S. Ryu J. Y. Kim S. Y. Jeon J. H. Song J. Y. Cho H. T.. (2007). Transcriptional regulation of the respiratory genes in the cyanobacterium Synechocystis sp. Pcc 6803 during the early response to glucose feeding. Plant Physiol. 145, 1018–1030. doi: 10.1104/pp.107.105023, PMID: 17827271 PMC2048796

[ref65] Liu L.-N. Bryan S. J. Huang F. Yu J. Nixon P. J. Rich P. R.. (2012). Control of electron transport routes through redox-regulated redistribution of respiratory complexes. Proc. Natl. Acad. Sci. 109, 11431–11436. doi: 10.1073/pnas.1120960109, PMID: 22733774 PMC3396541

[ref66] Lu K.-J. Chang C.-W. Wang C.-H. Chen F. Y. H. Huang I. Y. Huang P.-H.. (2023). An Atp-sensitive phosphoketolase regulates carbon fixation in cyanobacteria. Nat. Metab. 5, 1111–1126. doi: 10.1038/s42255-023-00831-w, PMID: 37349485 PMC10365998

[ref67] Luque I. Flores E. Herrero A. (1994). Molecular mechanism for the operation of nitrogen control in cyanobacteria. EMBO J. 13, 2862–2869. doi: 10.1002/j.1460-2075.1994.tb06580.x, PMID: 8026471 PMC395167

[ref68] Marbach D. Costello J. C. Küffner R. Vega N. M. Prill R. J. Camacho D. M.. (2012). Wisdom of crowds for robust gene network inference. Nat. Methods 9, 796–804. doi: 10.1038/nmeth.2016, PMID: 22796662 PMC3512113

[ref69] Markson S. Piechura J. R. Puszynska A. M. O’Shea E. K. (2013). Circadian control of global gene expression by the cyanobacterial master regulator RpaA. Cell 155, 1396–1408. doi: 10.1016/j.cell.2013.11.005, PMID: 24315105 PMC3935230

[ref70] Maus I. Koeck D. E. Cibis K. G. Hahnke S. Kim Y. S. Langer T.. (2016). Unraveling the microbiome of a thermophilic biogas plant by metagenome and metatranscriptome analysis complemented by characterization of bacterial and archaeal isolates. Biotechnol. Biofuels 9:581. doi: 10.1186/s13068-016-0581-3, PMID: 27525040 PMC4982221

[ref71] Miao X. Wu Q. Wu G. Zhao N. (2003). Sucrose accumulation in salt-stressed cells of agp gene deletion-mutant in cyanobacterium Synechocystis sp Pcc 6803. FEMS Microbiol. Lett. 218, 71–77. doi: 10.1111/j.1574-6968.2003.tb11500.x 12583900

[ref72] Mistry J. Chuguransky S. Williams L. Qureshi M. Salazar G. A. Sonnhammer E. L. L.. (2021). Pfam: The protein families database in 2021. Nucleic Acids Res. 49, D412–D419. doi: 10.1093/nar/gkaa913, PMID: 33125078 PMC7779014

[ref73] Monteiro P. T. Pedreira T. Galocha M. Teixeira M. C. Chaouiya C. (2020). Assessing regulatory features of the current transcriptional network of *Saccharomyces cerevisiae*. Sci. Rep. 10:17744. doi: 10.1038/s41598-020-74043-7, PMID: 33082399 PMC7575604

[ref74] Morby A. P. Turner J. S. Huckle J. W. Robinson N. J. (1993). SmtB is a metal-dependent repressor of the cyanobacterial metallothionein gene smtA: identification of a Zn inhibited Dna-protein complex. Nucleic Acids Res. 21, 921–925. doi: 10.1093/nar/21.4.921, PMID: 8451191 PMC309225

[ref75] Mullineaux C. W. (2014). Electron transport and light-harvesting switches in cyanobacteria. Front. Plant Sci. 5:7. doi: 10.3389/fpls.2014.00007, PMID: 24478787 PMC3896814

[ref76] Nakahira Y. Katayama M. Miyashita H. Kutsuna S. Iwasaki H. Oyama T.. (2004). Global gene repression by KaiC as a master process of prokaryotic circadian system. Proc. Natl. Acad. Sci. U. S. A. 101, 881–885. doi: 10.1073/pnas.0307411100, PMID: 14709675 PMC321775

[ref77] Nakamura K. Hihara Y. (2006). Photon flux density-dependent gene expression in *Synechocystis* sp. Pcc 6803 is regulated by a small, redox-responsive, LuxR-type regulator*. J. Biol. Chem. 281, 36758–36766. doi: 10.1074/jbc.M606797200, PMID: 17035238

[ref78] Naseri A. Sharghi M. Hasheminejad S. M. H. (2021). Enhancing gene regulatory networks inference through hub-based data integration. Comput. Biol. Chem. 95:107589. doi: 10.1016/j.compbiolchem.2021.107589, PMID: 34673384

[ref79] Newman M. E. (2008). *The mathematics of networks*. The new palgrave encyclopedia of economics, no. 2, pp. 1–12.

[ref80] Nishiwaki T. Satomi Y. Nakajima M. Lee C. Kiyohara R. Kageyama H.. (2004). Role of KaiC phosphorylation in the circadian clock system of *Synechococcus elongatus* Pcc 7942. Proc. Natl. Acad. Sci. 101, 13927–13932. doi: 10.1073/pnas.0403906101, PMID: 15347812 PMC518855

[ref81] Nordberg H. Cantor M. Dusheyko S. Hua S. Poliakov A. Shabalov I.. (2014). The genome portal of the Department of Energy Joint Genome Institute: 2014 updates. Nucleic Acids Res. 42, D26–D31. doi: 10.1093/nar/gkt1069, PMID: 24225321 PMC3965075

[ref82] Ortet P. De Luca G. Whitworth D. E. Barakat M. (2012). P2tf: a comprehensive resource for analysis of prokaryotic transcription factors. BMC Genomics 13:628. doi: 10.1186/1471-2164-13-628, PMID: 23153078 PMC3532121

[ref83] Park K. Kim D. (2009). Localized network centrality and essentiality in the yeast-protein interaction network. Proteomics 9, 5143–5154. doi: 10.1002/pmic.200900357, PMID: 19771559

[ref84] Passemiers A. Moreau Y. Raimondi D. Mathelier A. (2022). Fast and accurate inference of gene regulatory networks through robust precision matrix estimation. Bioinformatics 38, 2802–2809. doi: 10.1093/bioinformatics/btac178, PMID: 35561176 PMC9113237

[ref85] Patel A. Mcgrosso D. Hefner Y. Campeau A. Sastry A. V. Maurya S.. (2023). Proteome allocation is linked to transcriptional regulation through a modularized transcriptome. Nat. Commun. 15:5234. doi: 10.1038/s41467-024-49231-y PMC11187210 38898010

[ref86] Piechura J. R. Amarnath K. O'shea E. K. (2017). Natural changes in light interact with circadian regulation at promoters to control gene expression in cyanobacteria. eLife 6:32. doi: 10.7554/eLife.32032, PMID: 29239721 PMC5785211

[ref87] Pliakos K. Vens C. (2019). Network inference with ensembles of bi-clustering trees. BMC Bioinformatics 20:525. doi: 10.1186/s12859-019-3104-y, PMID: 31660848 PMC6819564

[ref88] Puszynska A. M. O'shea E. K. (2017). Switching of metabolic programs in response to light availability is an essential function of the cyanobacterial circadian output pathway. eLife 6:210. doi: 10.7554/eLife.23210, PMID: 28430105 PMC5400509

[ref89] Razaghi-Moghadam Z. Nikoloski Z. (2020). Supervised learning of gene-regulatory networks based on graph distance profiles of transcriptomics data. NPJ Syst. Biol. Appl. 6:21. doi: 10.1038/s41540-020-0140-1, PMID: 32606380 PMC7327016

[ref90] Riediger M. Kadowaki T. Nagayama R. Georg J. Hihara Y. Hess W. R. (2019). Biocomputational analyses and experimental validation identify the regulon controlled by the redox-responsive transcription factor RpaB. iScience 15, 316–331. doi: 10.1016/j.isci.2019.04.033, PMID: 31103851 PMC6525291

[ref91] Saito M. Watanabe S. Nimura-Matsune K. Yoshikawa H. Nakamoto H. (2020). Regulation of the groesl1 transcription by the HrcA repressor and a novel transcription factor Orf7.5 in the cyanobacterium *Synechococcus elongatus* Pcc7942. J. Gen. Appl. Microbiol. 66, 85–92. doi: 10.2323/jgam.2020.02.001, PMID: 32281544

[ref92] Salgado H. Gama-Castro S. Lara P. Mejia-Almonte C. Alarcón-Carranza G. López-Almazo A. G.. (2024). Regulondb v12.0: a comprehensive resource of transcriptional regulation in *E. coli* K-12. Nucleic Acids Res. 52, D255–D264. doi: 10.1093/nar/gkad1072, PMID: 37971353 PMC10767902

[ref93] Santos-Merino M. Sakkos J. K. Singh A. K. Ducat D. C. (2024). Coordination of carbon partitioning and photosynthesis by a two-component signaling network in *Synechococcus elongatus* Pcc 7942. Metab. Eng. 81, 38–52. doi: 10.1016/j.ymben.2023.11.001, PMID: 37925065

[ref94] Schumann C. Fernández Méndez J. Berggren G. Lindblad P. (2023). Novel concepts and engineering strategies for heterologous expression of efficient hydrogenases in photosynthetic microorganisms. Front. Microbiol. 14:607. doi: 10.3389/fmicb.2023.1179607, PMID: 37502399 PMC10369191

[ref95] Schwarz D. Nodop A. Hüge J. Purfürst S. Forchhammer K. Michel K. P.. (2011). Metabolic and transcriptomic phenotyping of inorganic carbon acclimation in the cyanobacterium *Synechococcus elongatus* Pcc 7942. Plant Physiol. 155, 1640–1655. doi: 10.1104/pp.110.170225, PMID: 21282404 PMC3091134

[ref96] Seki A. Hanaoka M. Akimoto Y. Masuda S. Iwasaki H. Tanaka K. (2007). Induction of a group 2 σ factor, Rpod3, by high light and the underlying mechanism in *Synechococcus elongatus* Pcc 7942. J. Biol. Chem. 282, 36887–36894. doi: 10.1074/jbc.M707582200, PMID: 17977831

[ref97] Shin J. Zielinski D. C. Palsson B. O. (2024). Deciphering nutritional stress responses via knowledge-enriched transcriptomics for microbial engineering. Metab. Eng. 84, 34–47. doi: 10.1016/j.ymben.2024.05.007, PMID: 38825177

[ref98] Shinde S. Zhang X. Singapuri S. P. Kalra I. Liu X. Morgan-Kiss R. M.. (2020). Glycogen metabolism supports photosynthesis start through the oxidative pentose phosphate pathway in Cyanobacteria. Plant Physiol. 182, 507–517. doi: 10.1104/pp.19.01184, PMID: 31649110 PMC6945877

[ref99] Sierro N. Makita Y. De Hoon M. Nakai K. (2008). Dbtbs: a database of transcriptional regulation in *Bacillus subtilis* containing upstream intergenic conservation information. Nucleic Acids Res. 36, D93–D96. doi: 10.1093/nar/gkm910, PMID: 17962296 PMC2247474

[ref100] Sorrells T. R. Johnson A. D. (2015). Making sense of transcription networks. Cell 161, 714–723. doi: 10.1016/j.cell.2015.04.014, PMID: 25957680 PMC4531093

[ref101] Taniguchi Y. Takai N. Katayama M. Kondo T. Oyama T. (2010). Three major output pathways from the Kaiabc-based oscillator cooperate to generate robust circadian kaibc expression in cyanobacteria. Proc. Natl. Acad. Sci. 107, 3263–3268. doi: 10.1073/pnas.0909924107, PMID: 20133618 PMC2840301

[ref102] Tatusov R. L. Natale D. A. Garkavtsev I. V. Tatusova T. A. Shankavaram U. T. Rao B. S.. (2001). The cog database: new developments in phylogenetic classification of proteins from complete genomes. Nucleic Acids Res. 29, 22–28. doi: 10.1093/nar/29.1.22, PMID: 11125040 PMC29819

[ref103] Teixeira M. C. Monteiro P. T. Palma M. Costa C. Godinho C. P. Pais P.. (2018). Yeastract: an upgraded database for the analysis of transcription regulatory networks in *Saccharomyces cerevisiae*. Nucleic Acids Res. 46, D348–d353. doi: 10.1093/nar/gkx842, PMID: 29036684 PMC5753369

[ref104] Teixeira M. C. Viana R. Palma M. Oliveira J. Galocha M. Mota M. N.. (2023). Yeastract+: a portal for the exploitation of global transcription regulation and metabolic model data in yeast biotechnology and pathogenesis. Nucleic Acids Res. 51, D785–D791. doi: 10.1093/nar/gkac1041, PMID: 36350610 PMC9825512

[ref105] The UniProt Consortium (2023). UniProt: the universal protein knowledgebase in 2023. Nucleic Acids Res. 51, D523–D531. doi: 10.1093/nar/gkac1052, PMID: 36408920 PMC9825514

[ref106] Tierrafría V. H. Rioualen C. Salgado H. Lara P. Gama-Castro S. Lally P.. (2022). Regulondb 11.0: comprehensive high-throughput datasets on transcriptional regulation in *Escherichia coli* K-12. Microb. Genom. 8:mgen000833. doi: 10.1099/mgen.0.000833, PMID: 35584008 PMC9465075

[ref107] Vijayan V. Zuzow R. O'shea E. K. (2009). Oscillations in supercoiling drive circadian gene expression in cyanobacteria. Proc. Natl. Acad. Sci. 106, 22564–22568. doi: 10.1073/pnas.0912673106, PMID: 20018699 PMC2799730

[ref108] Virtanen P. Gommers R. Oliphant T. E. Haberland M. Reddy T. Cournapeau D.. (2020). SciPy 1.0: fundamental algorithms for scientific computing in Python. Nat. Methods 17, 261–272. doi: 10.1038/s41592-019-0686-2, PMID: 32015543 PMC7056644

[ref109] Welkie D. G. Rubin B. E. Diamond S. Hood R. D. Savage D. F. Golden S. S. (2019). A hard Day’s night: *Cyanobacteria* in diel cycles. Trends Microbiol. 27, 231–242. doi: 10.1016/j.tim.2018.11.002, PMID: 30527541 PMC6377297

[ref110] Wilson D. Charoensawan V. Kummerfeld S. K. Teichmann S. A. (2008). Dbd––taxonomically broad transcription factor predictions: new content and functionality. Nucleic Acids Res. 36, D88–D92. doi: 10.1093/nar/gkm964, PMID: 18073188 PMC2238844

[ref111] Winterbach W. Mieghem P. V. Reinders M. Wang H. Ridder D. D. (2013). Topology of molecular interaction networks. BMC Syst. Biol. 7:90. doi: 10.1186/1752-0509-7-90, PMID: 24041013 PMC4231395

[ref112] Woelfle M. A. Xu Y. Qin X. Johnson C. H. (2007). Circadian rhythms of superhelical status of DNA in cyanobacteria. Proc. Natl. Acad. Sci. USA 104, 18819–18824. doi: 10.1073/pnas.0706069104, PMID: 18000054 PMC2141860

[ref113] Xu Y. Mori T. Johnson C. H. (2000). Circadian clock-protein expression in cyanobacteria: rhythms and phase setting. EMBO J. 19, 3349–3357. doi: 10.1093/emboj/19.13.3349, PMID: 10880447 PMC313937

[ref114] Xu Y. Mori T. Pattanayek R. Pattanayek S. Egli M. Johnson C. H. (2004). Identification of key phosphorylation sites in the circadian clock protein KaiC by crystallographic and mutagenetic analyses. Proc. Natl. Acad. Sci. 101, 13933–13938. doi: 10.1073/pnas.0404768101, PMID: 15347809 PMC518856

[ref115] Yilmaz S. Nyerges A. Van Der Oost J. Church G. M. Claassens N. J. (2022). Towards next-generation cell factories by rational genome-scale engineering. Nat. Catal. 5, 751–765. doi: 10.1038/s41929-022-00836-w

[ref116] Yousef N. Pistorius E. K. Michel K. P. (2003). Comparative analysis of idiA and isiA transcription under iron starvation and oxidative stress in *Synechococcus elongatus* Pcc 7942 wild-type and selected mutants. Arch. Microbiol. 180, 471–483. doi: 10.1007/s00203-003-0618-4, PMID: 14605795

[ref117] Zhao M. He W. Tang J. Zou Q. Guo F. (2021). A comprehensive overview and critical evaluation of gene regulatory network inference technologies. Brief. Bioinform. 22:9. doi: 10.1093/bib/bbab009, PMID: 33539514

[ref118] Zorro-Aranda A. Escorcia-Rodríguez J. M. González-Kise J. K. Freyre-González J. A. (2022). Curation, inference, and assessment of a globally reconstructed gene regulatory network for *Streptomyces coelicolor*. Sci. Rep. 12:2840. doi: 10.1038/s41598-022-06658-x 35181703 PMC8857197

